# Machine learning algorithms for a novel cuproptosis-related gene signature of diagnostic and immune infiltration in endometriosis

**DOI:** 10.1038/s41598-023-48990-w

**Published:** 2023-12-07

**Authors:** Jiajia Wang, Yiming Lu, Guangyu Sun, Zhihao Fang, Zhiyong Xing, Weihua Nong, Yunbao Wei, Shan Wang, Guiling Shi, Mingyou Dong, Junli Wang

**Affiliations:** 1 Department of Obstetrics and Gynecology, The Affiliated Hospital of Youjiang Medical University for Nationalities, Baise, 533000 China; 2 Graduate School of Youjiang, Medical University for Nationalities, Baise, 533000 China; 3 Chaozhou People’s Hospital, Shantou University Medical College, Chaozhou, 515600 China; 4 School of Medical Laboratory, Youjiang Medical University for Nationalities, Baise, 533000 China; 5 Department of Stomatology, The Affiliated Hospital of Youjiang Medical University for Nationalities, Baise, 533000 China; 6 Department of Laboratory Medicine, The Affiliated Hospital of Youjiang Medical University for Nationalities, Baise, 533000 China

**Keywords:** Cancer, Computational biology and bioinformatics

## Abstract

Endometriosis (EMT) is an aggressive disease of the reproductive system, also called “benign cancer”. However, effective treatments for EMT are still lacking in clinical practice. Interestingly, immune infiltration is significantly involved in EMT pathogenesis. Currently, no studies have shown the involvement of cuproptosis-related genes (CRGs) in regulating immune infiltration in EMT. This study identified three CRGs such as GLS, NFE2L2, and PDHA1, associated with EMT using machine learning algorithms. These three CRGs were upregulated in the endometrium of patients with moderate/severe EMT and downregulated in patients with infertility. Single sample genomic enrichment analysis (ssGSEA) revealed that these CRGs were closely correlated with autoimmune diseases such as systemic lupus erythematosus. Furthermore, these CRGs were correlated with immune cells such as eosinophils, natural killer cells, and macrophages. Therefore, profiling patients based on these genes aid in a more accurate diagnosis of EMT progression. The mRNA and protein expression levels of GLS, NFE2L2 and PDHA1 were validated by qRT-PCR and WB studies in EMT samples. These findings provide a new idea for the pathology and treatment of endometriosis, suggesting that CRGs such as GLS, NFE2L2 and PDHA1 may play a key role in the occurrence and development of endometriosis.

## Introduction

Endometriosis (EMT) is a common estrogen-dependent disease wherein the endometrial tissue implants and grows on the peritoneal surface, ovaries, rectovaginal septum, perineum, and surgical scars^1^. The primary symptoms of EMT include lower abdominal pain, dysmenorrhea, menstrual abnormalities, and discomfort during intercourse. Globally, nearly 5–10% of women of reproductive age are affected by EMT, and 30–50% of women with EMT suffer from infertility^2,3^. However, the pathogenesis of EMT has not yet been elucidated. Several studies have identified markers associated with EMT^4^. However, the sample size of these studies was small, which prevents accurate diagnosis. Therefore, there is an urgent need to identify new biomarkers to understand the pathophysiological mechanisms of EMT. This would aid in designing better and more accurate diagnostic as well as therapeutic approaches for EMT.

Copper is an essential cofactor regulating several enzymes involved in various cellular functions, including mitochondrial respiration, antioxidation, and synthesis of hormones, neurotransmitters, and pigment. However, abnormal copper levels can induce oxidative stress and cytotoxicity^5^. Recent studies have demonstrated that copper induces cell death by altering the tricarboxylic acid cycle (TCA), which causes the accumulation of lipid-acylated proteins and a decrease in iron-sulfur cluster protein levels. This triggers a proteotoxic stress response called cuproptosis^6^. Moreover, studies have demonstrated that copper homeostasis is critically involved in immune cell infiltration. Tan et al. showed that copper-induced H2O2 production mediated by LOXL4 activates the type I interferon signaling pathway, which promotes PD-L1 presentation by macrophages^7^. A study has demonstrated a close correlation between the copper-related protein STEAP2 and the prognosis, as well as the infiltration of immune cells in gliomas^8^. Ji et al. indicated that cuproptosis could influence the development and prognosis of patients with renal clear cell carcinoma by regulating immune cell infiltration^9^.

Therefore, it is necessary to study the involvement of cuproptosis in the diagnosis and immune cell infiltration in diseases. However, no studies have determined the influence of cuproptosis-mediated immune cell infiltration on EMT pathogenesis. Therefore, in this study, we combined data on EMTs and cuproptosis-related genes (CRGs) to determine the underlying mechanism by which cuproptosis mediates immune cell infiltration in EMT. First, we obtained data on 19 CRGs from previous studies^10^ and the GSE7307 datasets from the "Gene Expression Omnibus (GEO)” database to perform differential and functional correlation analysis. Next, we identified three key CRGs using machine learning algorithms. Furthermore, we analyzed the correlation between key CRGs, immune cells, and pathways. Finally, we constructed a nomogram and screened for drug targets to provide insights into cuproptosis in EMT. The outcomes of this study are summarized in the following table. These results would aid in enhancing our understanding of the involvement of cuproptosis in regulating EMT.

## Material and methods

### Data download and pre-processing

The gene expression data set analyzed in this study came from the GEO database (http://www.ncbi.nlm.nih.gov/geo/). The search criteria used the keyword "Endometriosis" and selected the organism "Homo sapiens" from the dataset platform GPL570 [HG-U133_Plus_2] Affymetrix Human Genome U133 Plus 2.0 Array. The number of samples in the control group and disease group of the GEO data set are both greater than 10 and are finally included in the data set 7307 and GSE7305 for subsequent analysis^11–16^. We use the GSE7307 data set as the training set and GSE7307 as the validation set. In this study, we first searched the GEO database (https://www.ncbi.nlm.nih.gov/geo/) and downloaded the gene expression profiles of endometriosis (GSE7307 and GSE7305) using the "GEOQuery" package of R software (version 4.1.2). GSE7307 dataset consisting of 677 samples, however, only 18 endometriosis and 23 normal endometrium was used as a train set. Based on the same GSE7307 platform, GSE7305, from the GEO database as a validation cohort. This dataset consisting of ten ovarian endometriosis and ten normal endometrium. None of the samples were on medication or had received hormone therapy prior to surgery. In order to understand the expression of key CRGs in EMT with different severities and combined with infertility, we downloaded GSE51981 and GSE120103. GSE51981 and GSE120103 data sets are based on the GPL570 and GPL6480 Agilent-014850 Whole Human Genome Microarray 4 × 44 K G4112F (Probe Name version) platforms for further verification^17^. Gene expression profiles were normalized using the normal Between Arrays function of the Limma package, and method = "scale". Meanwhile, 6 EMT samples were collected from EMT patients, and 6 normal endometrium samples were collected from women who underwent hysterectomy for uterine fibroids. All participants signed an informed consent form. The study was approved by the Institutional Research Ethics Committees of the Affiliated Hospital of Youjiang Medical University for Nationalities (YYFY-LL-2023-137) and conformed to the guidelines and regulations stated in the Declaration of Helsinki.

### Screening for differentially expressed CRGs

In order to find out the differences in the expression of cuproptosis-related genes (CRGs) in EMT patients, after downloading the gene chip analysis data, we normalized the gene expression and CRGs were then screened from the GSE7307 and GSE7305 datasets using the “LIMMA “ package of R software. CR-DEGS screening was performed on 28 EMT samples and 33 control endometrial tissues using P < 0.05 and |logFC|> 0.2 as the criteria for screening. The ”Ggpubr “ package of R is used to display heatmaps and box plots of the results.

### Functional enrichment Analysis of DEGs

We used the “Disease Ontology (DO),” “Gene Ontology (GO),” and “Kyoto Encyclopedia of Genes and Genomes (KEGG) pathway enrichment analyses” to determine the biological roles of CRGs. P < 0.05 was considered statistically significant. Further, the visual enrichment maps were constructed using the "ggplot2" and "GOplot" R packages.

### Machine learning algorithm to identify key CRGs

Three machine learning algorithms; the least absolute shrinkage and selection operator (LASSO), Random Forest (RF)^18^ and Support Vector Machines-RFE (SVM-RFE)^19^ were used to screen feature genes. Initial assessment of EMT differentially expressed CRGs using LASSO. Furthermore, the value of the penalty parameter (λ) filters out the tenfold cross-validation with the lowest deviation anomaly probability. After regression analysis, only genes with non-zero coefficients were retained. Use the random forest algorithm for additional screening of key genes and filter variables with a relative importance greater than 0.5. SVM-RFE was used to further screen the characteristic genes, and the top 6 genes with average ranking were retained for subsequent analysis. LASSO, RF and SVM-RFE were crossed to obtain our signature genes. Finally, the results were visualized by constructing a Venn diagram using the "venneuler" R package.

### Diagnostic value of key CRGs in EMT and external validation

We determined the diagnostic value of these key CRGs in GSE7307 and validated these genes in GSE7305. For GSE51981 and GSE120103, we plotted the receiver operating characteristic (ROC) curves to determine the diagnostic value of these genes using “P < 0.05” as the threshold. We calculated the area under the ROC curve (AUC), and the AUC value between 0 and 1 was used for analysis. The greater the AUC value, the better the performance of these genes in predicting diagnosis.

### Screening key regulatory pathways using Single sample genomic enrichment analysis (ssGSEA)

A set of 16 immune cell and 13 immune-related pathway^20^ was obtained from references. Take these 29 gene sets and the gene expression matrix as input files and run a single-sample gene set enrichment analysis (ssGSEA) on all samples using "gsva" R package^21^. The infiltration rates of 16 immune cell types and the activities of 13 immune system-related signaling pathways were calculated.

### Immunological analysis

The relative proportions of the 22 types of infiltrating immune cells in all EMT samples were determined using “Cell-type Identification by Estimating Relative Subsets of RNA Transcripts” (CIBERSORT, http://cibersortx.stanford.edu). Next, we calculated immune scores using the “Estimation of Stromal and Immune cells in Malignant Tumor tissues using Expression data" algorithm. Finally, Spearman rank correlation analysis was performed to determine the correlation between CRGs and the number of infiltrating immune cells. The correlation was visualized using the “ggplot2” package.

### Construction of nomogram prediction model and efficacy assessment for clinical diagnosis of EMT

We constructed a nomogram prediction model and validated it internally using the "rms" R package. The risk score is calculated based on the expression of individual key genes, and the total risk score is defined as the sum of the risk scores of all individual genes. The performance of the model was assessed using calibration curves, clinical decision curve analysis (DCA), and ROC curves.

### Drug screening for key CRGs

The “DrugBank” database (https://go.drugbank.com/drugs) was used to predict potential therapeutic targets of key CRGs associated with EMT. The “DrugBank database is a unique bioinformatics and cheminformatics resource that contains more than 13,791 drug entries, including small molecules, biotechnology, nutraceuticals, and investigational drugs. Additionally, there are > 5236 nonredundant protein sequences associated with these drug entries.

### Quantitative RealTime polymerase chain reaction (qRT-PCR)

Total RNA was isolated from EMT using Trizol reagent. The cDNA was then synthesized by reverse transcription using the RevertAid First Strand cDNA Synthesis Kit, and Real-time PCR was performed on these cDNAs using Fast SYBR Green PCR Master Mix. The qRT-PCR FRG primers used in this study were specified in Supplementary Table 1.

### Western blotting

The total protein quantity from CC were lysed with RIPA lysate, electrophoresed by SDS-PAGE and transferred to PVDF membranes, then the membranes were closed with 5% skim milk powder for 2 h. Primary antibodies GLS (1:1000, abcam, UK), NFE2L2 (1:1000, abcam, UK) and PDHA1 (1:1000, abcam, UK) were incubated overnight at 4 °C and secondary antibodies were incubated at room temperature for 2 h. Bands were detected with ECL chromogenic solution, and GAPDH was used as a control for analysis.

### Statistical analysis

R 4.0.5 statistical software was used to complete the analysis. We used the t-test and Wilcoxon rank-sum test for quantifying variables. Furthermore, Spearman correlation analysis was used to investigate the correlation between CRGs and immune cell infiltration. P < 0.05 was considered statistically significant.

## Results

### Screening for DEGs in patients with EMT

**Figure 1:**
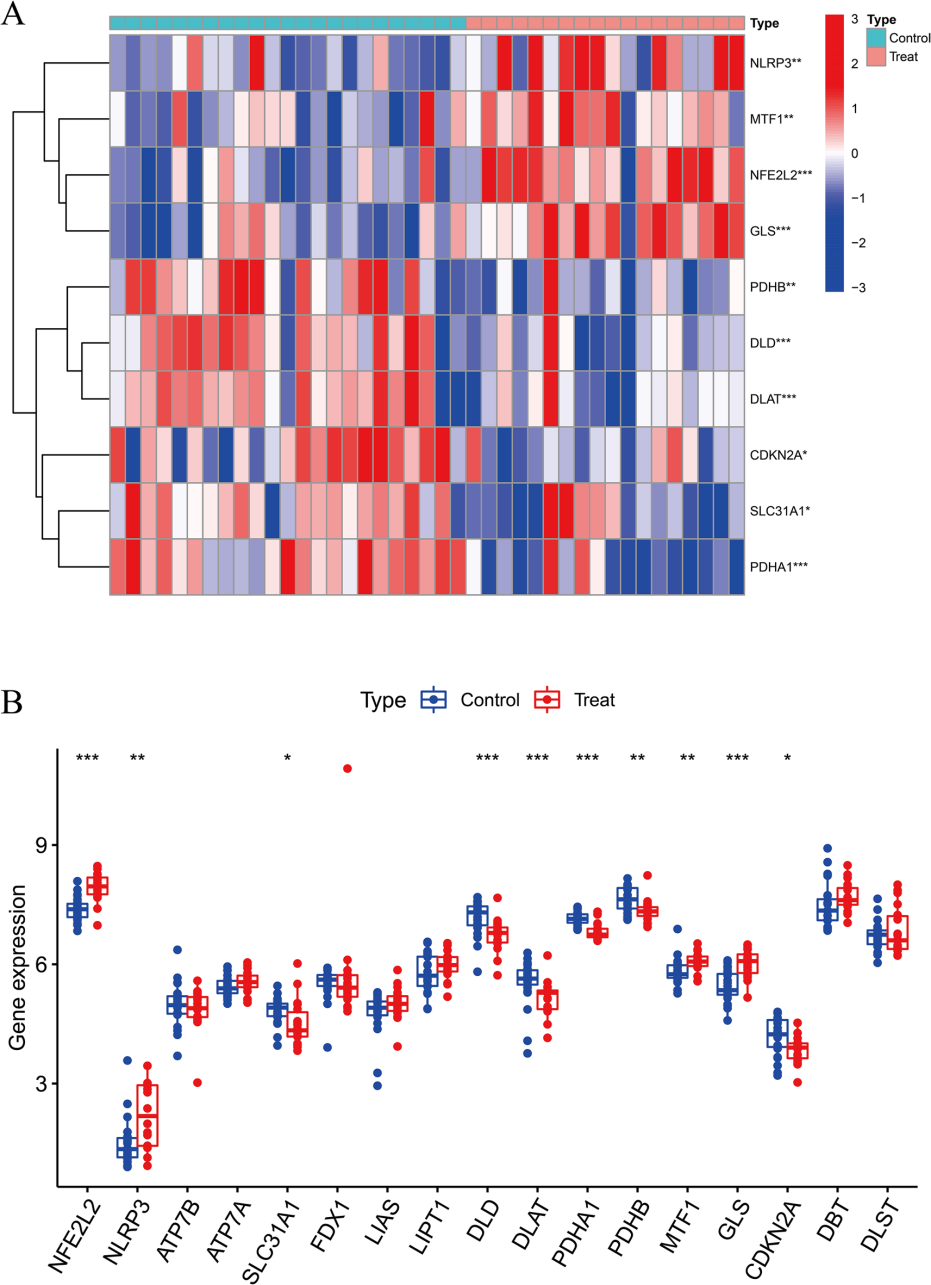
The dysregulated genes were shown in the **A** heat map via analyzing GSE7307 datasets. 10 CRGs were identified between EMT and normal samples. **B** CRGs expression in EMT and normal samples, blue: normal samples, red: tumor samples (*p < 0.05, **p < 0.01, ***p < 0.001).

After downloading the gene chip analytic data, we normalized the gene expression and the data are shown in Supplementary Fig. S1. We combined 19 CRGs from previously published studies and DEGs screened from patients with EMT. A total of 10 DEGs were screened from GSE7307, of which six were upregulated, and four were downregulated (Fig. 1A, B).

### Functional enrichment analysis of DEGs

**Figure 2:**
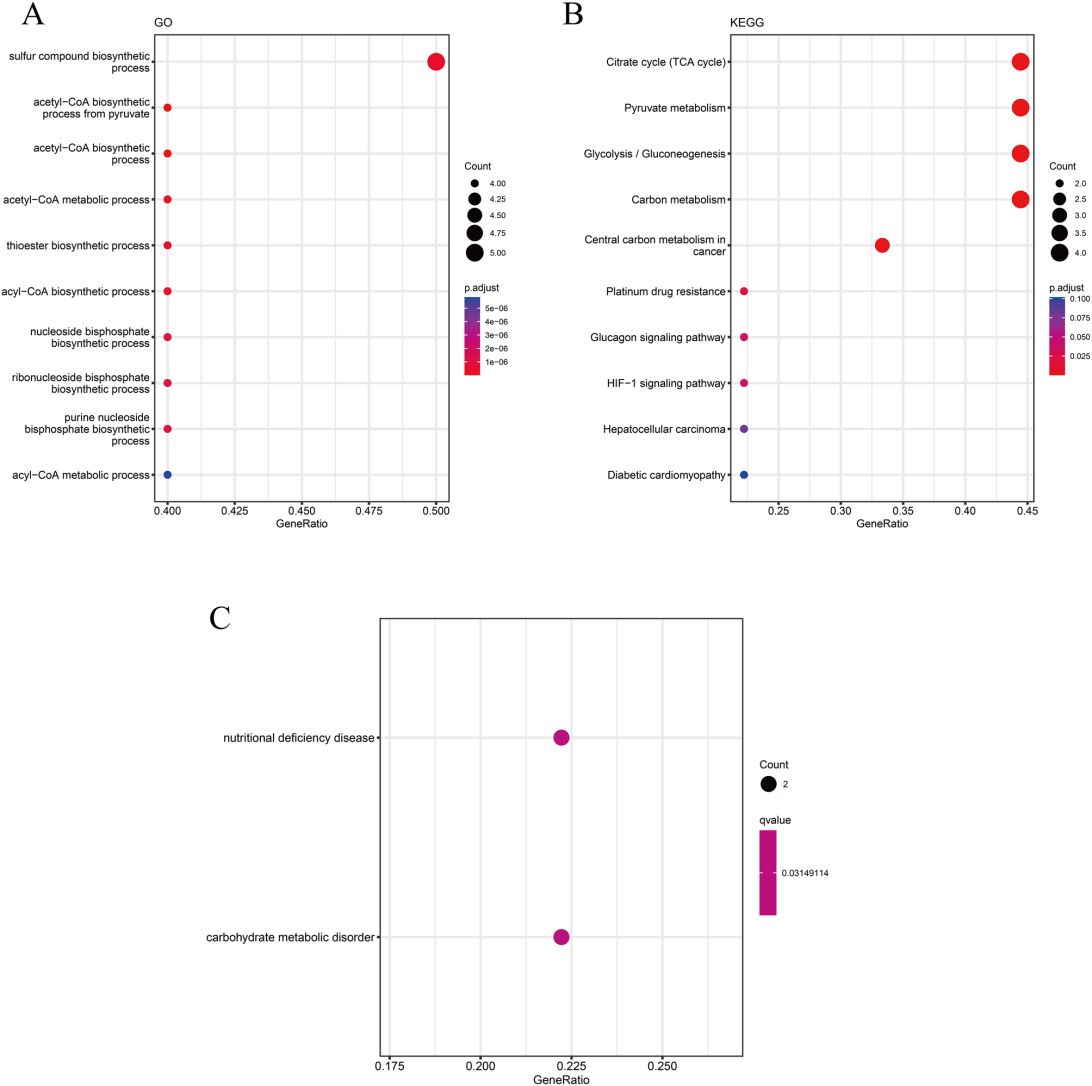
**A** DO enrichment analysis of 10 CRGs in EMT. **B** GO enrichment analysis of 10 CRGs in EMT. **C** KEGG pathway analyses of 10 CRGs in EMT.

We performed the DO, GO, and KEGG pathway enrichment analyses on DEGs. The DO enrichment analysis demonstrated that these key CRGs were mainly enriched in the nutrient deficiency disease pathway associated with carbohydrate metabolism disorder (Fig. 2A). The GO enrichment analysis revealed that these key CRGs were enriched in various processes such as acetyl-CoA biosynthesis from pyruvate, acetyl-CoA metabolism, and thioester, acyl-a CoA, a sulfur compound, nucleoside bisphosphate, ribonucleoside, and acyl-CoA biosynthesis processes (Fig. 2B). The KEGG enrichment analysis showed pathways, including TCA, Glycolysis/Gluconeogenesis, pyruvate, and carbon metabolism, as well as central carbon metabolism in cancer, resistance to platinum drugs, and the glucagon and HIF-1 signaling pathways were enriched by these CRGs. However, no significant difference was observed between these CRGs (Fig. 2C).

### Screening of EMT markers using machine learning algorithms

**Figure 3:**
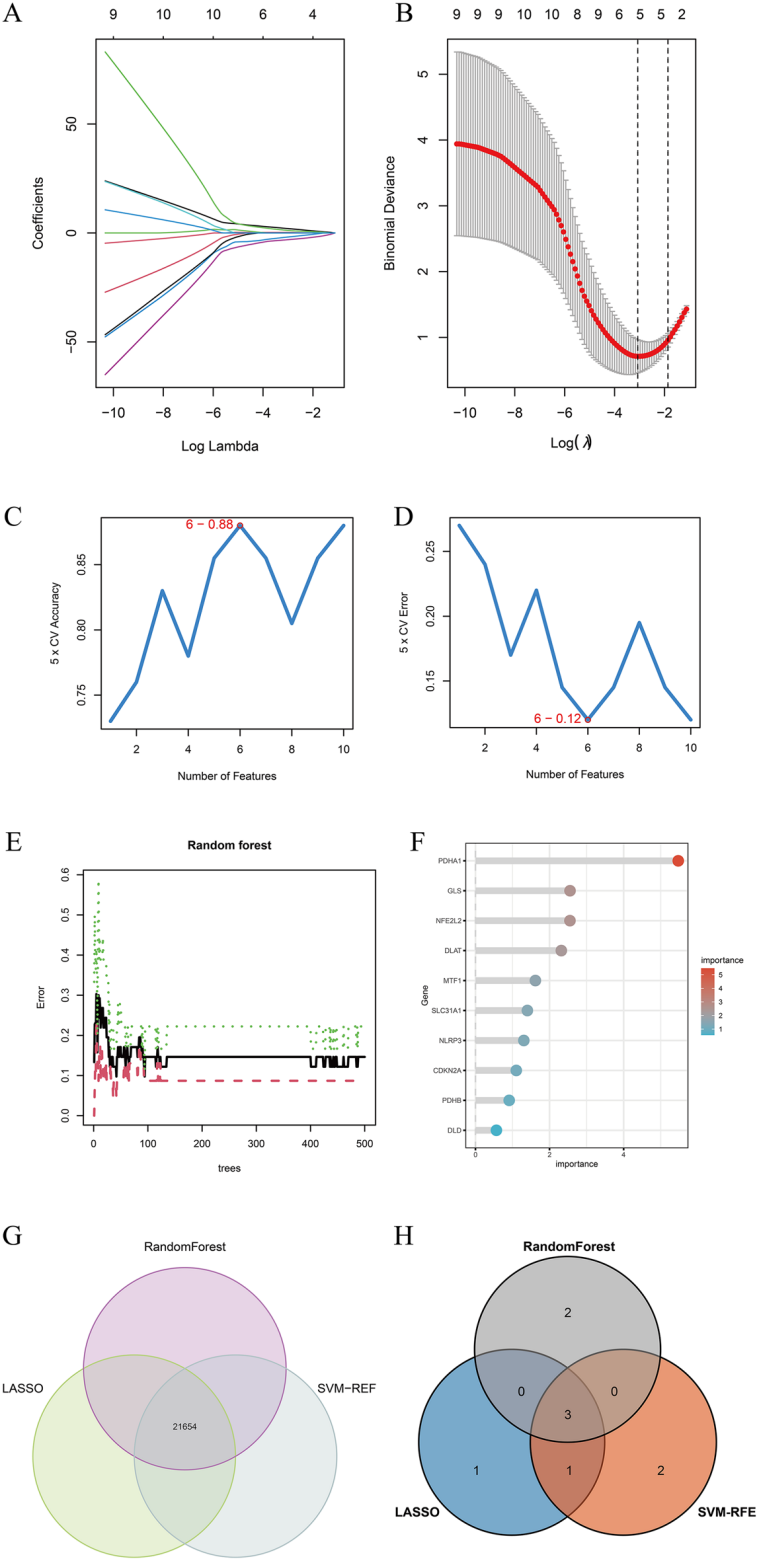
Selection procedure of diagnostic markers for EMT diagnoses. (**A, B**) Tuning feature selection in the LASSO model. (**C, D**) Biomarker signature gene expression validation through the SVM-RFE arithmetic. (**E**) RandomForest error rate versus the number of classification trees. **F** The top 10 relatively important genes. (**G, H**) Venn graph presenting 3 diagnostic biomarkers shared by the LASSO, SVM-RFE and RandomForest arithmetic methods.

A total of ten DEGs were identified using machine learning as signature genes. Five signature genes, including NFE2L2, NLRP3, DLD, PDHA1, and GLS, were identified using the “LASSO regression analysis” (Fig. 3A, B). Next, six signature genes, including GLS, PDHB, DLD, NFE2L2, PDHA1, and SLC31A1, were identified using the “SVM-RFE” algorithm (Fig. 3C, D). Finally, five signature genes, including PDHA1, GLS, NFE2L2, DLAT, and MTF1, were identified using the “RF” algorithm (Fig. 3E, F). Further, genes identified using these three machine learning algorithms were intersected, and three signature genes, including GLS, NFE2L2, and PDHA1, were identified. The three genes were intersected to obtain three signature genes, GLS, NFE2L2, and PDHA1, and plotted on a Wayne diagram (Fig. 3G, H).

### Expression and validation of the diagnostic significance of the signature genes

**Figure 4:**
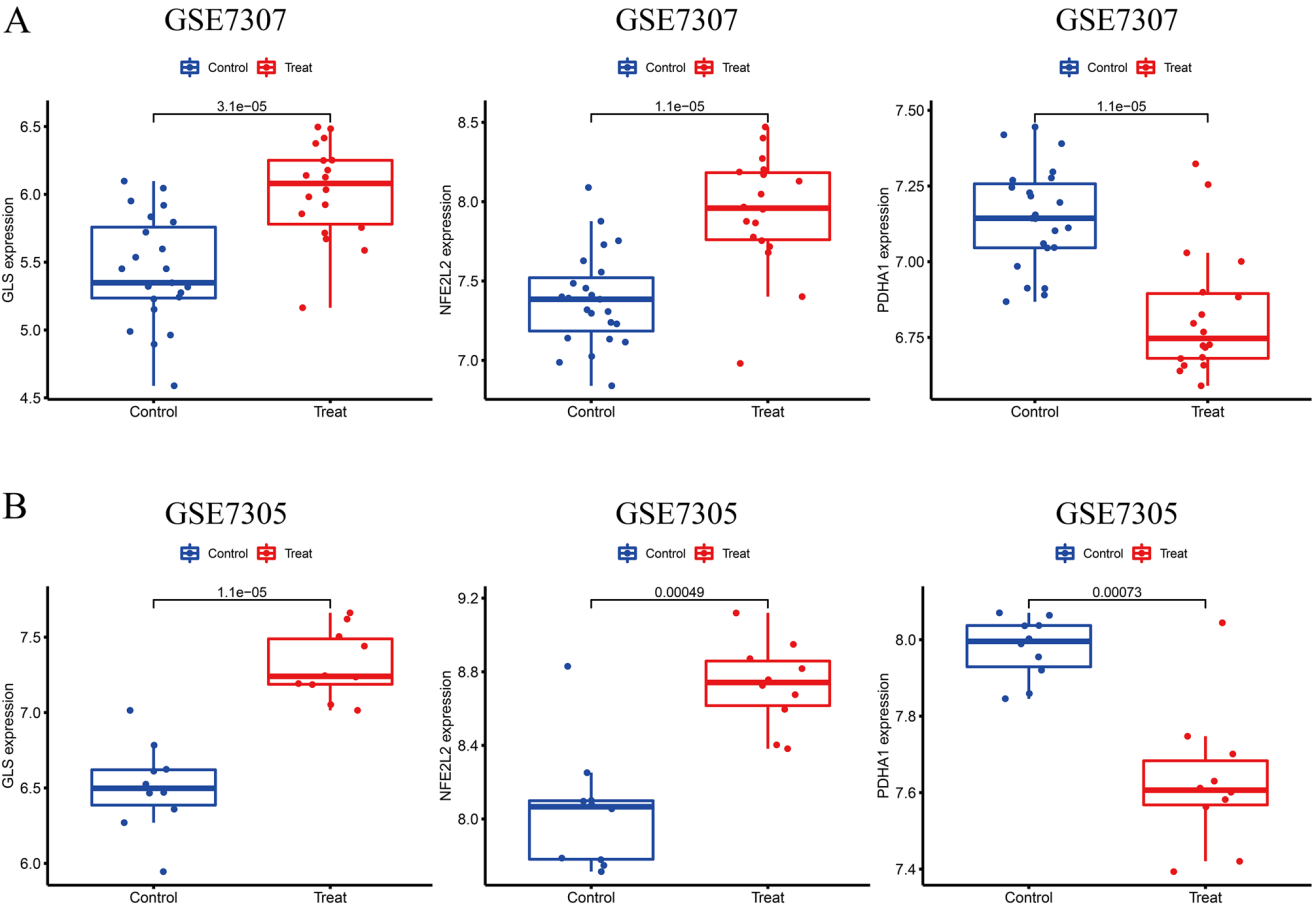
The expression pattern of the 3 critical genes in EMT. **A** GLS and NFE2L2 were highly expressed in EMT, and PDHA1 was lowly expressed in EMT. **B** The expression pattern of the 3 critical genes was further demonstrated in GSE7305 datasets.

**Figure 5:**
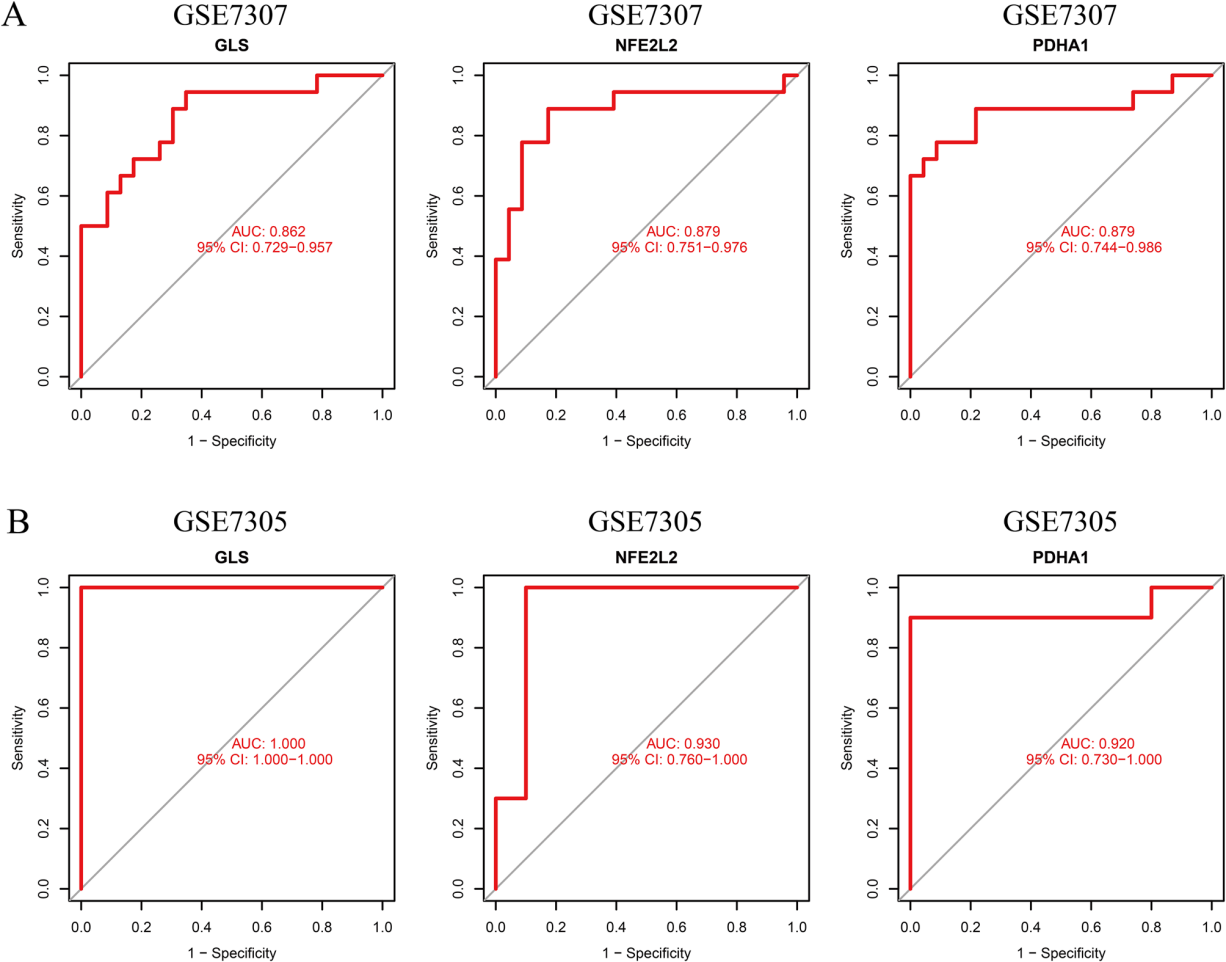
**A** The diagnostic value of the 3 critical genes was studied using ROC assays in GSE7307. **B** Three genes (GLS, NFE2L2, and PDHA1) were further demonstrated to be diagnostic genes in GSE7305.

First, we determined the differential expression and diagnostic value of GLS, NFE2L2, and PDHA1 in GSE7307. Further, these genes were externally validated in GSE7305. The results revealed a significant increase in GLS and NFE2L2 expression and a significant decrease in PDHA1 expression in both datasets (Fig. 4A, B). The AUC values of PDHA1 in the two datasets were 0.879 and 0.920, respectively (Fig. 5A, B). The AUC values of all three diagnostic genes were > 0.800, thereby indicating these CRGs had high diagnostic values.

### Expression of key CRGs in EMT with different severity and combined infertility

**Figure 6:**
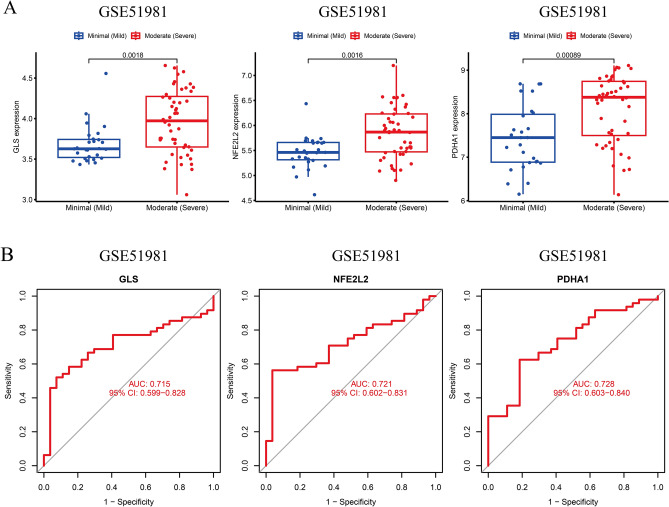
The expression pattern of the 3 critical genes in different severity groups of endometrioses. **A** Expression of CRGs in different severity groups of endometrioses. **B** ROC analysis of CRGs predicting the severity of endometriosis in GSE51981.

**Figure 7:**
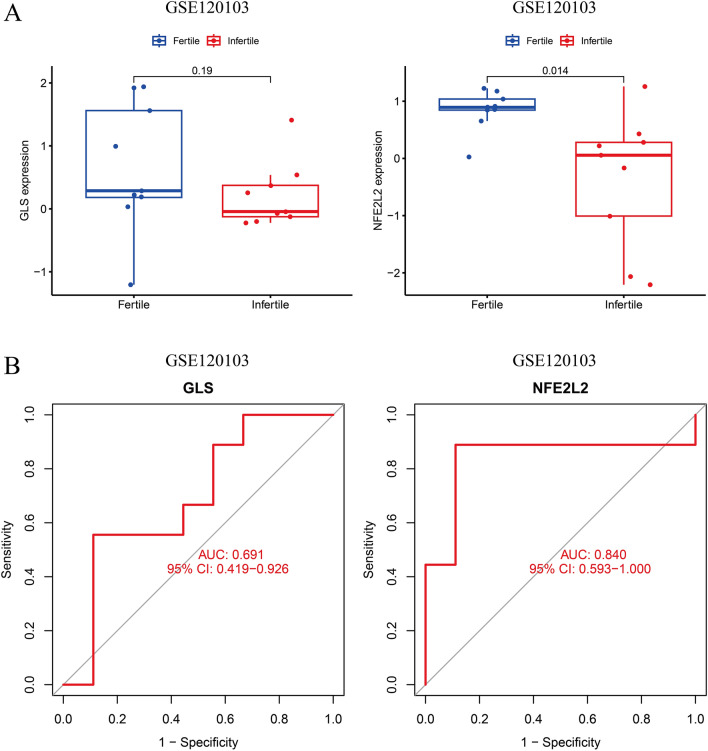
Expression of CRGs in the endometria of the infertile and fertile females with endometriosis. **A** Upregulated CRGs were decreased in the infertile group. **B** The ROC analysis of CRGs predicting infertility of endometriosis in GSE120103.

To validate the confidence and applicability of the key CRGs, external datasets such as GSE51981 and GSE120103 were selected to validate the correlation between key CRGs, EMT severity, and combined infertility. Since PDA1 was not expressed in GSE120103, PDHA1 was not analyzed in EMT samples. The patients with micro/light EMT were categorized into one group, and patients with moderate/severe EMT were categorized into another group. The results revealed high GLS and NFE2L2 expression in patients with moderate/severe EMT (Fig. 6A). ROC curves were performed on these upregulated key CRGs to analyze the severity of EMT (P < 0.05) and the AUC values > 0.7 (Fig. 6B). Next, we used GSE120103, consisting of patients with EMT who may or may not be suffering from infertility, for subsequent validation. The results revealed an increase in GLS and NFE2L2 expression in the endometrium of patients with EMT; however, a decrease in GLS and NFE2L2 expression was observed in the endometrium of patients with infertility (Fig. 7A). On the contrary, we performed ROC analysis to determine the significance of key CRGs in diagnosing patients with EMT. The AUC values validated the sensitivity and specificity of CRGs (Fig. 7B).

### Analyzing Regulatory pathway enriched by key CRGs

**Figure 8:**
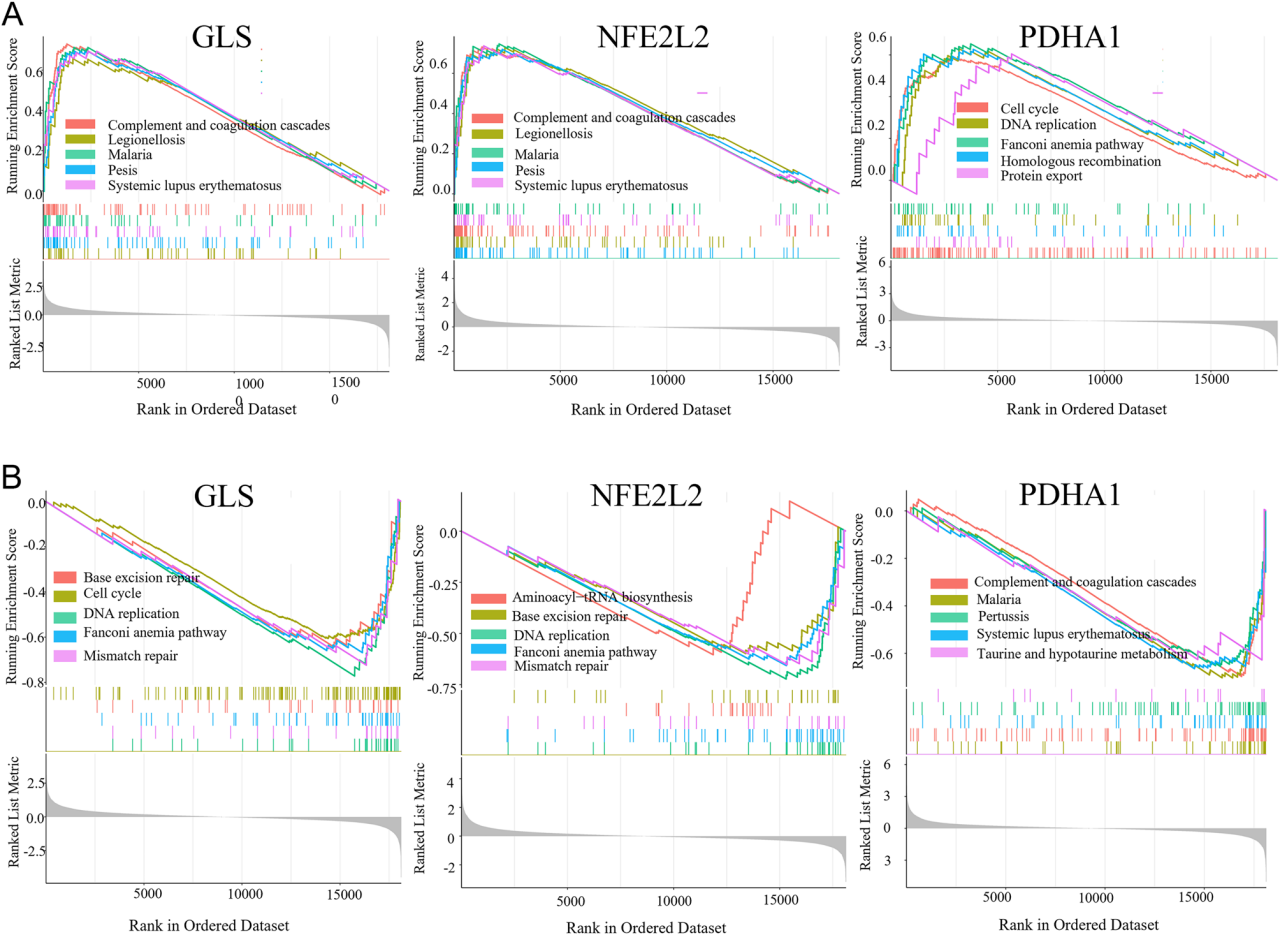
GSEA revealed the enriched pathways of the hub genes. **A** The main signaling pathways that are significantly enriched by the high expression of GLS, NFE2L2 and PDHA1. **B** The main signaling pathways that are significantly enriched by the low expression of key CRGs.

The ssGSEA results showed that the complement and coagulation pathways, Legionellosis, Malaria, Pertussis, Systemic lupus erythematosus (SLE), etc., were enriched in the high GLS and high NFE2L2 expression groups (Fig. 8A, B). In addition, the cell cycle, the Fanconi anemia and protein export pathways, DNA replication, and Homologous recombination are enriched in the PDHA1 expression group (Fig. 8C).

### Correlation between three key CRGs and proportion of immune cell infiltration in EMT

**Figure 9:**
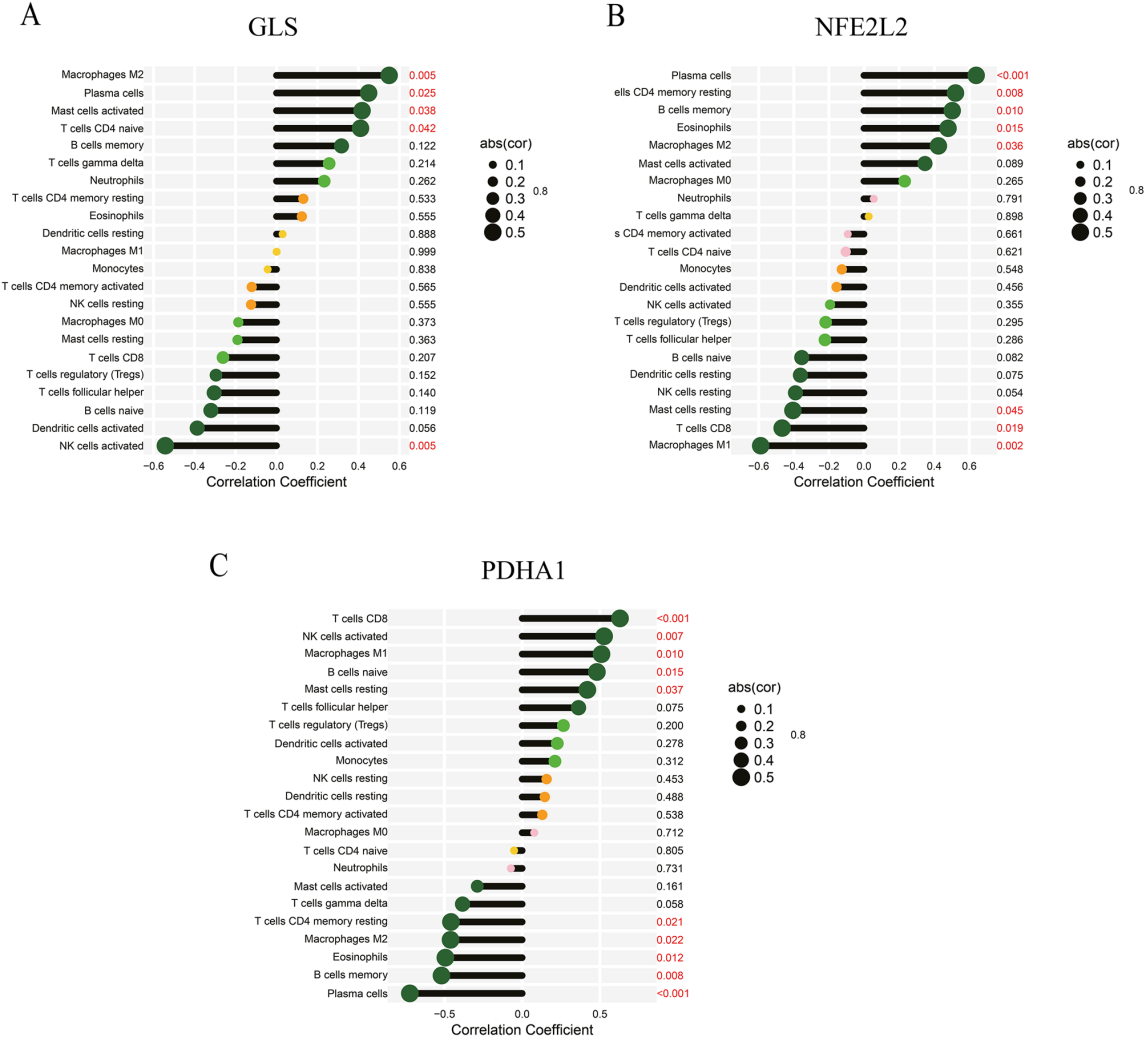
**A** Correlation between GLS and infiltrating immune cells in EMT. **B** Correlation between NFE2L2 and infiltrating immune cells in EMT. **C** Correlation between PDHA1 and infiltrating immune cells in EMT.

We utilized the “CIBERSORT” method to determine the proportion of 22 immune cell subpopulations infiltrating in patients with EMT. GLS, NFE2L2, and PDHA1 expression were evaluated as a function of the immune microenvironment. A significant positive correlation was observed between GLS and immune cells like M2 macrophages, Plasma cells, naïve CD4 T cells, and activated Mast cells. GLS was negatively correlated with activated NK cells (Fig. 9A). NFE2L2 was significantly correlated with immune cells such as Plasma cells, memory B cells, resting memory CD4 T cells, Eosinophils, M1 and M2 macrophages, resting mast cells, and CD8 T cells (Fig. 9B). Furthermore, a significant positive correlation was observed between NFE2L2 and immune cells such as activated NK cells, CD8T cells, M1 and M2 Macrophages, resting mast cells, naïve B cells, resting memory CD4 T cells, eosinophils, memory B cells, and Plasma cells (Fig. 9C).

### Construction and validation of the Nomogram prediction model

**Figure 10:**
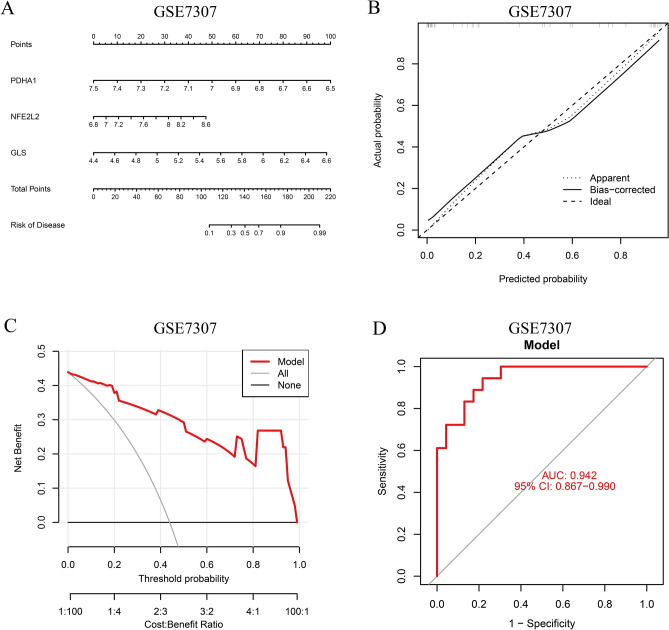
Construction and validation of the EMT diagnostic column line graph model. **A** Column line graphs are used to predict the occurrence of EMT. **B** Calibration curves to assess the predictive power of the column line graph model. **C** DCA curves to assess the clinical value of the column line graph model. **D** ROC curves to assess the clinical value of the column line graph model.

We constructed a nomogram prediction model using GLS, NFE2L2, and PDHA1 for predicting the risk of EMT development (Fig. 10A). Nomogram was externally validated using GSE7305. The rate of incidences was derived from each risk factor score and summed to obtain a total score. Next, we plotted the calibration curve (Fig. 10B). The results revealed that the three curves were consistent and converged, thereby indicating a good agreement between the actual and predicted outcomes of the model. In addition, DCA showed the clinical benefit of the nomogram prediction model (Fig. 10C). Finally, we plotted the ROC curve (the AUC value = 0.942) to determine the predictive ability of the columnar graph model (Fig. 10D). The AUC value of the nomogram prediction model was 0.942, thereby indicating that the model had a good predictive ability.

### Drug prediction

**Figure 11:**
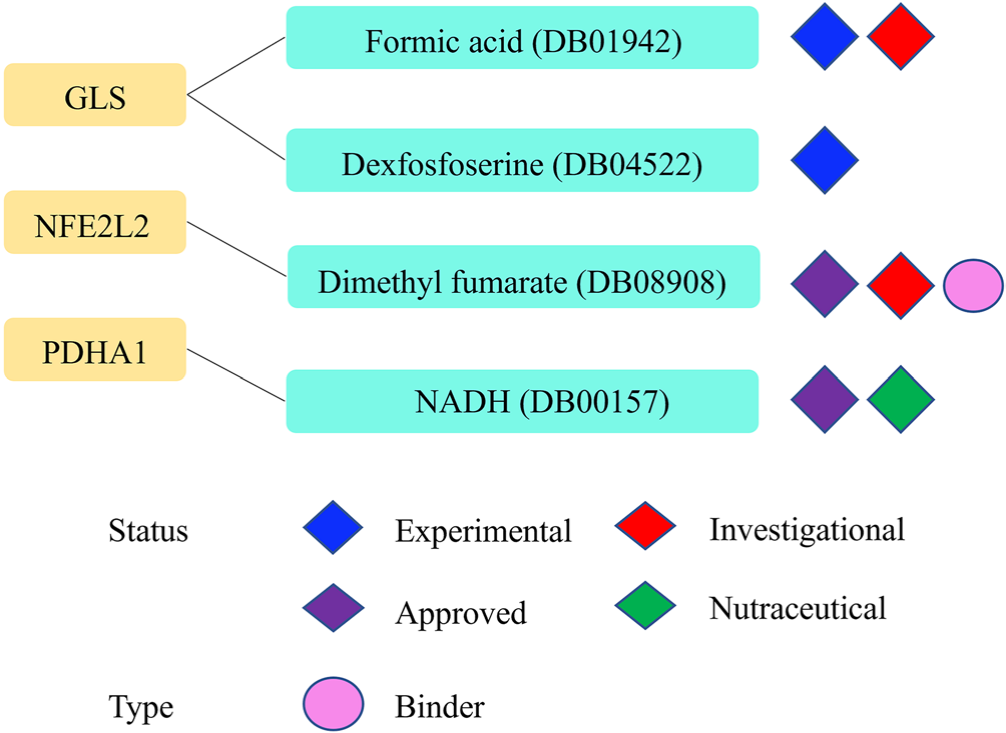
Drug screening for targeting key CRGs.

We identified five drugs targeting three key CRGs using data from the “DrugBank database (Fig. 11), and all of these drugs have been approved. First, L-glutamine (DB00130) was identified as a drug targeting CRGs. It is an important energy source in cells and is involved in metabolic processes. It is used to treat patients with sickle cell disease gastric and duodenal ulcers, improving brain function. Next, we identified Glutamate (DB00142), a common amino acid and an excitatory neurotransmitter. It is a precursor molecule required for GABA synthesis in GABAergic neurons. It is mainly used as total parenteral nutrition and as an adjunct for treating psycho-neurological disorders. Next, NADH (DB00157) targets PDHA1. Finally, dimethyl fumarate (DMF, DB08908) binds to NFE2L2. It is used for treating patients with relapsing multiple sclerosis.

### Validation of GLS, NFE2L2 and PDHA1 Expression in EMT by using qRT-PCR and Western blot

**Figure 12:**
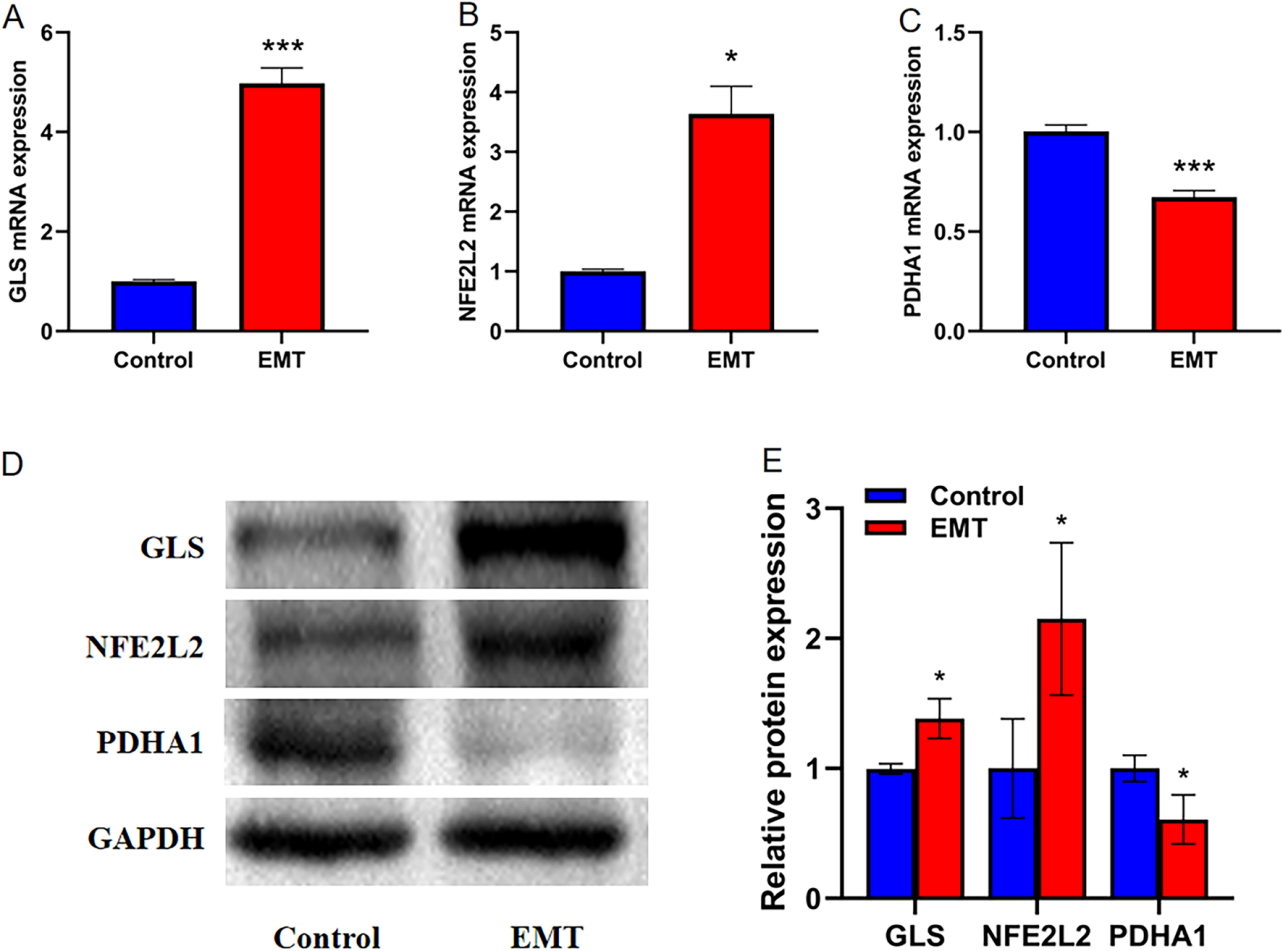
The correlation of key CRGs expression in EMT. **A** The levels of GLS, NFE2L2 and PDHA1 mRNA expression in EMT tissues. **B** The levels of GLS, NFE2L2 and PDHA1 protein expression in EMT.

The above analysis revealed that GLS, NFE2L2 and PDHA1 have the potential as prognostic biomarkers for EMT. We verified the expression of GLS, NFE2L2 and PDHA1 in EMT and normal endometrium using qRT-PCR and Western blot. qRT-PCR and Western blot showed that GLS and NFE2L2 were expressed at higher levels in EMT, and PDHA1 were expressed at lower levels in EMT (P < 0.05, Fig. 12A–E).

## Discussion

Currently, invasive techniques, such as laparoscopic histopathological examination, are used for EMT diagnosis. The most common treatment modalities include hormonal suppression or surgical excision/ablation of visible lesions. However, the therapeutic outcomes are unsatisfactory and often accompanied by adverse effects. Studies have identified several key molecules, such as adhesion molecules, matrix metalloproteinases, lncRNA HOTAIR, oxidative stress, and regulatory T cells, that are involved in EMT development^22,23^. However, the exact mechanism is still unclear. Therefore, there is an urgent need to identify new biomarkers associated with EMT pathogenesis. This would aid in developing non-invasive diagnostic and screening tools for patients with EMT.

Copper is an essential cofactor of enzymes. It is critically involved in various physiological processes and activities. Turgut et al. showed that copper was associated with the pathogenesis of EMT and oxidative stress^24^. Cuproptosis is a newly discovered form of cell death involving TCA regulation. Lipid-acylated proteins induce cytotoxic stress, which leads to cell death 6. However, no studies have shown the involvement of cuproptosis in EMT. Therefore, in this study, we combined data on EMT and CRGs from GEO. We identified 10 DEGs, including NFE2L2, NLRP3, SLC31A1, DLAT, DLD, PDHB, PDHA1, MTF1, CDKN2A, and GLS. The DO, GO, and KEGG pathway enrichment analysis showed that key CRGs were primarily enriched in the acetyl-CoA and pyruvate metabolism, TCA, and Glycolysis/Gluconeogenesis. Bradbeer et al. showed a correlation between TCA enzyme succinate dehydrogenase and EMT^25^. Interestingly, alteration in TCA occurs in cuproptosis, which induces cell death. Therefore, these results indicate that these CRGs could be a promising target for modulating cuproptosis in EMT, which could be a new therapeutic strategy for treating EMT.

Next, we used three machine learning algorithms, such as “LASSO regression,” “SVM-RFE,” and “RF,” to screen for EMT and cuproptosis-related biomarkers. We identified three CRGs associated with immune cell infiltration in EMT. Of these three CRGs, an increase in GLS and NFE2L2 expression and a decrease in PDHA1 expression were observed. Furthermore, the ROC curve analysis validated the diagnostic value of these key CRGs in distinguishing the endometrium of patients with EMT and healthy controls. Finally, we validated our results using the external validation dataset GSE7305, and the results obtained were consistent.

Glutaminase (GLS) is located on chromosome 2. It is a key enzyme mediating glutamine-to-glutamate conversion. GLS is converted to alpha-ketoglutarate, which enters TCA to produce energy. GLS is an oncogene, and an increase in GLS expression was observed in pancreatic ductal adenocarcinoma. Under oxidative stress, SUCLA2 increases glutamine catabolism and the production of nicotinamide adenine dinucleotide phosphate as well as glutathione by activating GLS, thereby ameliorating oxidative stress and promoting tumor growth^26^. Our results revealed a significant correlation between CRGs like GLS and EMT progression. Nuclear transcription factor E2-related factor 2 (NFE2 like bZIP transcription factor 2, NFE2L2, also known as Nrf2) is primarily localized in the cytoplasm. It is an antioxidant transcription factor that regulates inflammatory responses and oxidative stress in cells^27^. NFE2L2 variants are closely linked to the risk of human disease. Interestingly, oxidative damage promotes EMT progression. Previous studies have shown that NFE2L2 increases EMT risk by inhibiting processes such as oxidative stress against the growth and development of EMT^28,29^. The pyruvate dehydrogenase complex E1 alpha subunit (PDHA1) is the E1 subunit of the pyruvate dehydrogenase complex. It is primarily localized in the mitochondria and contains three serine residues, which could be activated by four inhibitory PDHK1-4 and two reactivated phosphatases (PDP1-2). These PDP1-2 are reversibly phosphorylated and regulate glycolysis and TCA^30,31^. Spakova et al. demonstrated that silencing HIF-1α/miR210 expression increases PDHA1 and decreases MITF-M expression. This promotes mitochondrial respiratory activity, which aids in eliminating reactive oxygen species in melanoma cells^32^. Our results indicate a significant role of GLS, NFE2L2 and PDHA1 in EMT pathogenesis. ROC curve analysis revealed that these key CRGs are significantly involved in the onset and progression of EMT, thereby indicating the potential diagnostic value of these CRGs in clinical settings. Moreover, risk modeling results suggested that GLS, NFE2L2 and PDHA1 could be risk factors for EMT. Based on previous studies and our results, CRGs may play an important role in EMT development, and the underlying mechanisms should be further investigated.

We also determined the expression of key CRGs in patients with varying severity of EMT and those suffering from infertility. The results revealed an increase in GLS and NFE2L2 expression in patients with moderate/severe EMT. Furthermore, an increase in the expression of key CRGs could lead to infertility in patients with EMT. Qiao et al. showed that isoniazid activates the Keap1/Nrf2 signaling pathway by inducing oxidative stress and apoptosis, thereby impairing the reproductive system and reducing fertility in mammals^33^. Ivanov et al. showed that melatonin could protect the developing embryo from oxidative stress by modulating NFE2L2, SOD1, and GPX1 expression^34^. These results indicate a close correlation between NFE2L2 and infertility, consistent with our results.

Next, we performed ssGSEA to verify the involvement of key CRGs in EMT. Our results revealed significant enrichment of the complement and coagulation pathways, Legionellosis, Malaria, Pertussis, and SLE in the high GLS and high NFE2L2 expression groups. On the contrary, the cell cycle, the Fanconi anemia and protein export pathways, DNA replication, and homologous recombination were enriched in the high PDHA1 expression group. SLE is an autoimmune disease characterized by the presence of specific autoantibodies, including antinuclear antibodies. Studies have shown a correlation between EMT and autoimmune diseases such as SLE and Sjögren's syndrome^35,36^.

However, the underlying mechanism of the pathogenesis of EMT is still unclear. Implantation of reflux menstrual blood, endocrine, genetic factors, angiogenesis, and stem cell differentiation are important factors associated with EMT pathogenesis. Of these factors, abnormal immune responses could be the underlying mechanism of EMT pathogenesis. In fact, the immune component of EMT has received widespread attention from researchers. Some studies have demonstrated that an increase in the secretion of proinflammatory cytokines due to immune dysfunction, impaired immune surveillance, and altered immune cell profiles contribute to EMT progression. Prolonged persistent immune dysregulation could lead to a state of chronic inflammation, thereby creating a conducive environment for promoting adhesion and angiogenesis. This could lead to a vicious cycle of EMT development and progression^37^. The “CIBERSORT” method is widely used to study EMT in humans. It is based on the principles of linear support vector regression to deconvolute the expression matrix of immune cell subtypes.

Therefore, we used CIBERSORT to assess infiltrating immune cells for determining the role of immune cell infiltration in EMT. Our results revealed that these three CRGs were associated with various immune cell types, including eosinophils, activated NK cells, and macrophages. Eidukaite et al. showed that epithelial cells in endometriotic foci secrete high levels of eosinophil-specific chemokines compared to in situ endometrial cells. These eosinophils and other myeloid cells enter the peritoneum, which triggers inflammatory and allergic responses^38^. Ścieżyńska et al. showed that endometriotic cells' survival and growth in the peritoneal cavity might also be due to their recognition and clearance by local immune cells such as macrophages and NK cells^39^. Ramírez-Pavez et al. demonstrated that macrophages accumulate in the peritoneal cavity of patients with EMT; however, their ability to clear migrating endometrial debris is reduced^40^. Furthermore, combining the results of differential immune cell infiltration and the correlation between cuproptosis and immune cell infiltration revealed that immune cells such as eosinophils, activated NK cells, and M2 Macrophages could be critically involved in the modulation of CRG-regulated immune function in EMT.

EMT is an aggressive disease. Koninckx et al. have suggested that oxidative stress and peritoneal microbiota due to retrograde menstruation could cause EMT. Therefore, pharmacological treatment could prevent new lesions and be prescribed post-surgery. Additionally, Sirohi et al. showed that inhibiting copper-induced toxicity increases the survival of endometriotic cells^41^. Therefore, copper chelators could be used as therapeutic agents for treating patients with EMT^42^. We identified five drugs from the DrugBank database targeting these three CRGs. Qinpi methicin (DB13155) has anti-inflammatory and antioxidant activities. Hence, qinpi methicin could be used for treating diseases caused by inflammation and oxidative stress, such as EMT. As-Sanie et al. demonstrated high forebrain insula glutamine (DB00130)-glutamate (DB00142) binding in EMT patients with chronic pelvic pain. High islet activity could cause chronic pelvic pain in patients with EMT; therefore, reducing islet glutamate levels could alleviate chronic pelvic pain^43^. DMF is an immunomodulatory and antioxidant molecule commonly used to treat patients with relapsing multiple sclerosis and psoriasis. It binds with NFE2L2. Chen et al. demonstrated that DMF, an Nrf2 agonist, could significantly influence immune responses and oxidative stress. Additionally, Yan et al. suggested that DMF could attenuate inflammation, oxidative stress, and iron death via the NRF2/ARE/NF-kB signaling pathway. Moreover, DMF improves cognitive dysfunction in rats with chronic hypoperfusion^44^. Also, EMT is closely associated with oxidative stress^45^. Therefore, it is necessary to investigate if DMF could be used for treating patients with EMT. NADH is a nutritional supplement. It has antioxidant properties, reduces anxiety, promotes neurotransmitter synthesis, and prevents dementia. Govatati et al. showed that altering the mitochondrial membrane complex I could be a risk factor for EMT. Mitochondrial membrane complex I catalyze the transfer of electrons from NADH to ubiquinone and is closely correlated with EMT, which could be a new therapeutic approach^46^.

The mRNA and protein expression levels of GLS, NFE2L2 and PDHA1 were validated by qRT-PCR and WB studies in EMT samples. However, our study has a few limitations. First, the sample size of our study was relatively small since data were only obtained from GEO. Therefore, additional studies using a larger sample size should be performed to validate our results. Second, although we have successfully identified three CRGs as potential biomarkers for immunophenotyping of EMT, no in vivo or in vitro studies were conducted to validate these results. Thus, future studies should focus on conducting in vivo or in vitro studies to validate these biomarkers.

## Conclusions

GLS, NFE2L2, and PDHA1 could be novel diagnostic markers for EMT, representing a significant breakthrough. Additionally, the correlation between these CRGs and immune cell infiltration could aid in developing effective immunotherapy for patients with EMT.

### Supplementary Information

Supplementary Information 1.

Supplementary Information 2.

Supplementary Information 3.

Supplementary Information 4.

Supplementary Information 5.

Supplementary Information 6.

Supplementary Information 7.

Supplementary Information 8.

Supplementary Information 9.

Supplementary Information 10.

Supplementary Information 11.

Supplementary Information 12.

Supplementary Information 13.

## Data Availability

Microarray data for human endometriosis and control specimens were obtained from the GEO database datasets GSE7307, GSE7305, GSE51981 and GSE120103.
