# Supplementary figures and images for: Machine learning algorithms for a novel cuproptosis-related gene signature of diagnostic and immune infiltration in endometriosis
Source: Sci Rep. 2023 Dec 7;13:21603. doi: 10.1038/s41598-023-48990-w (PMC10703883; doi:10.1038/s41598-023-48990-w)

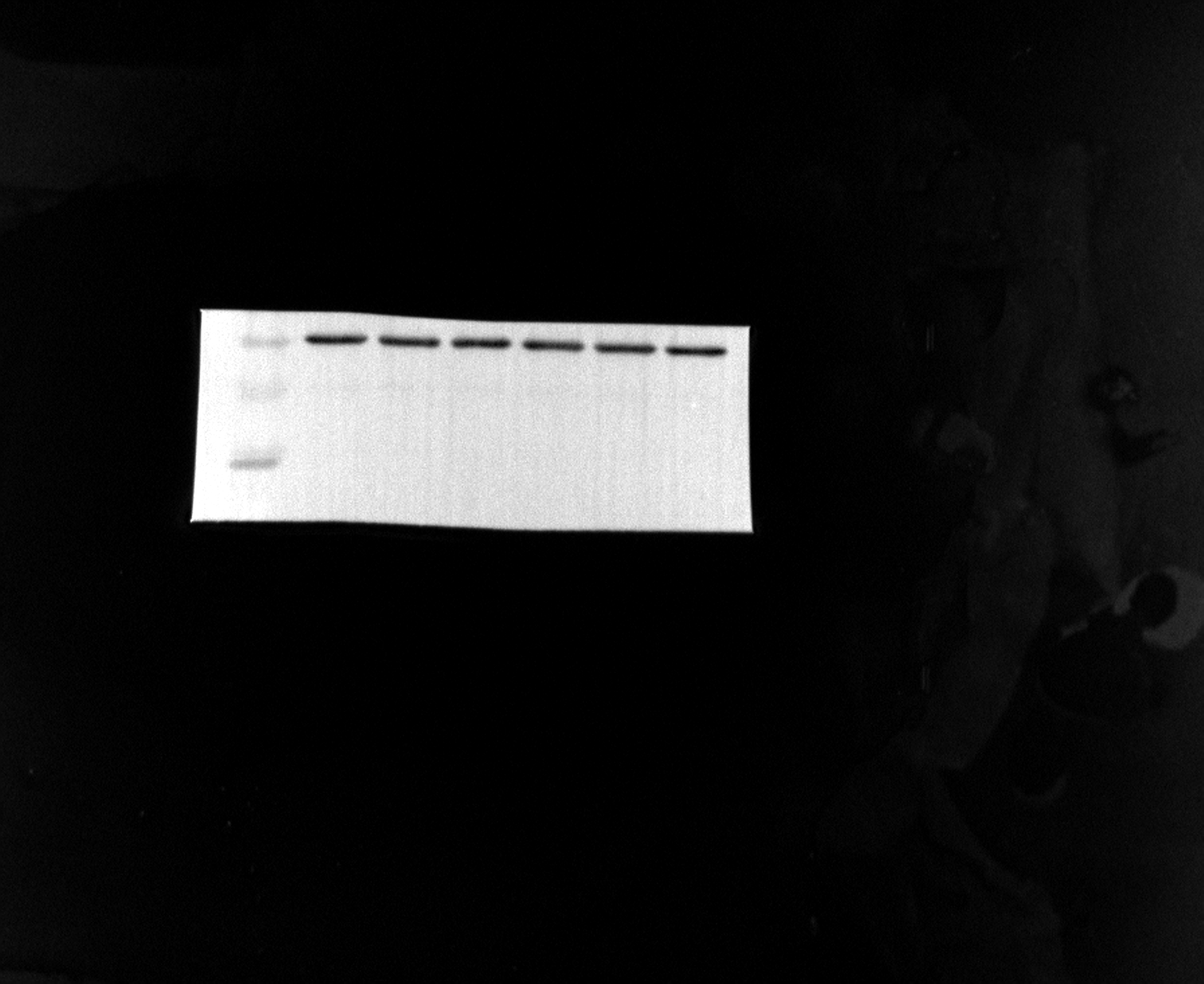

Supplement: Supplementary file 1 — Supplementary Information 1. [file 41598_2023_48990_MOESM1_ESM.tif]

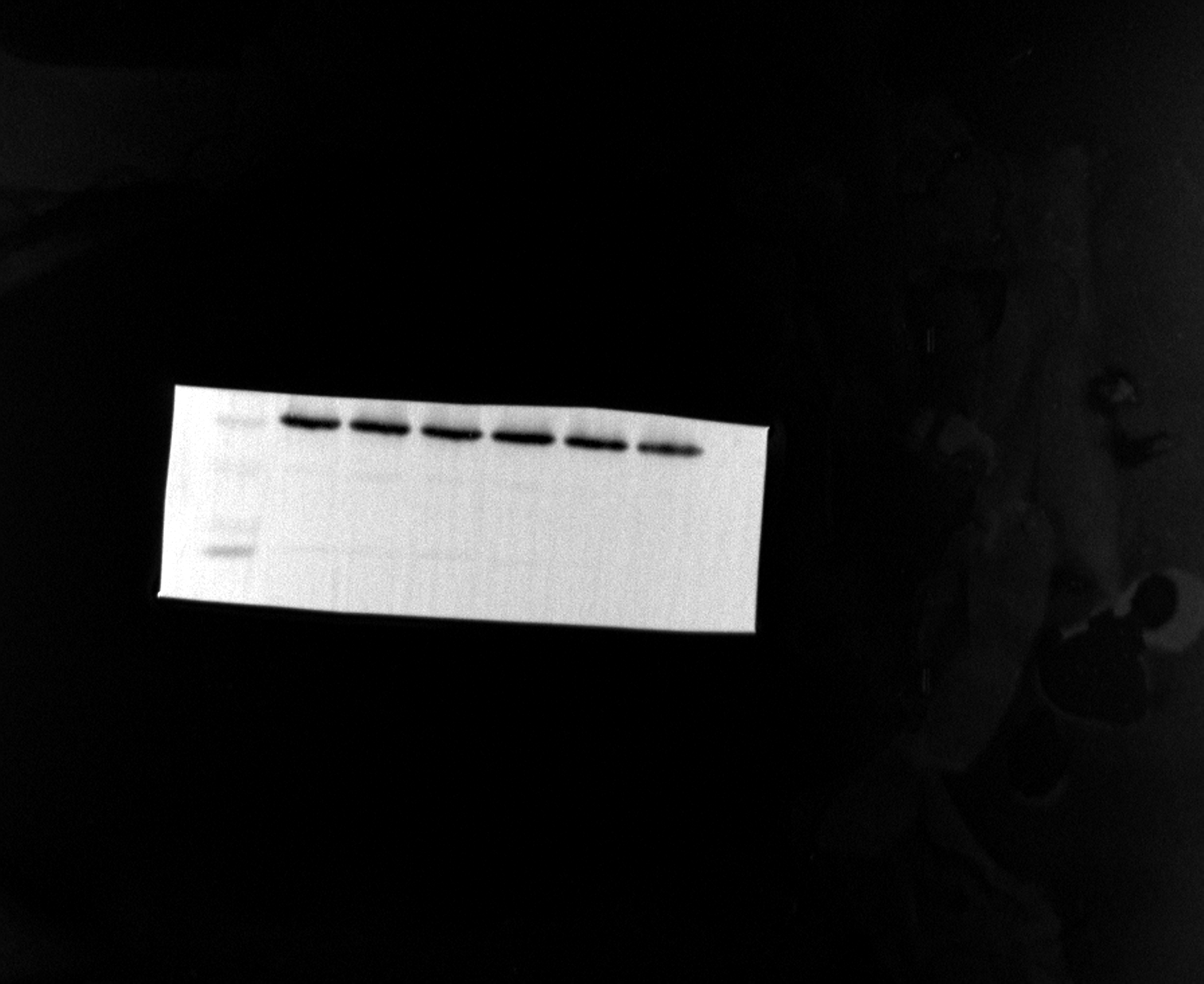

Supplement: Supplementary file 2 — Supplementary Information 2. [file 41598_2023_48990_MOESM2_ESM.tif]

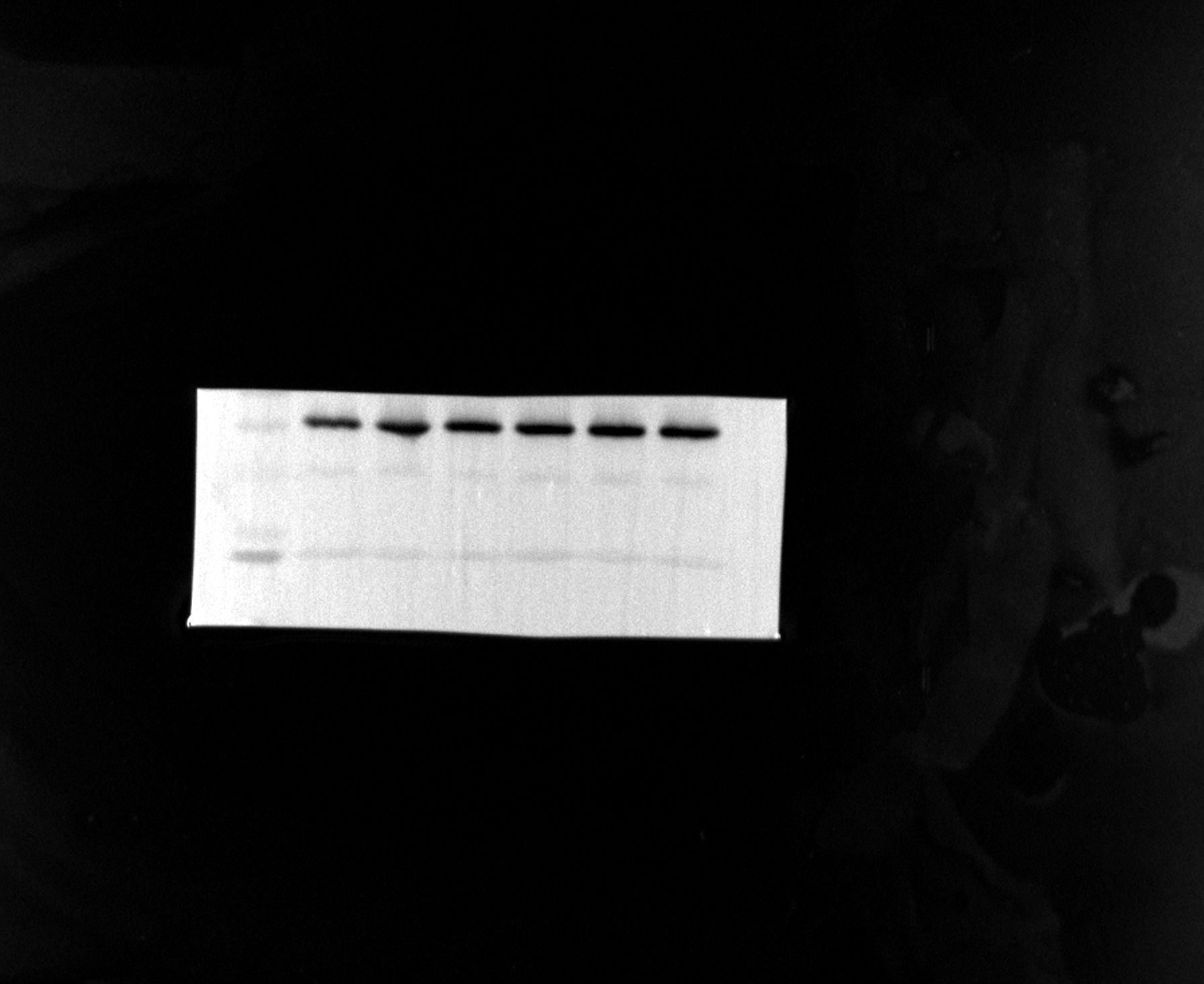

Supplement: Supplementary file 3 — Supplementary Information 3. [file 41598_2023_48990_MOESM3_ESM.tif]

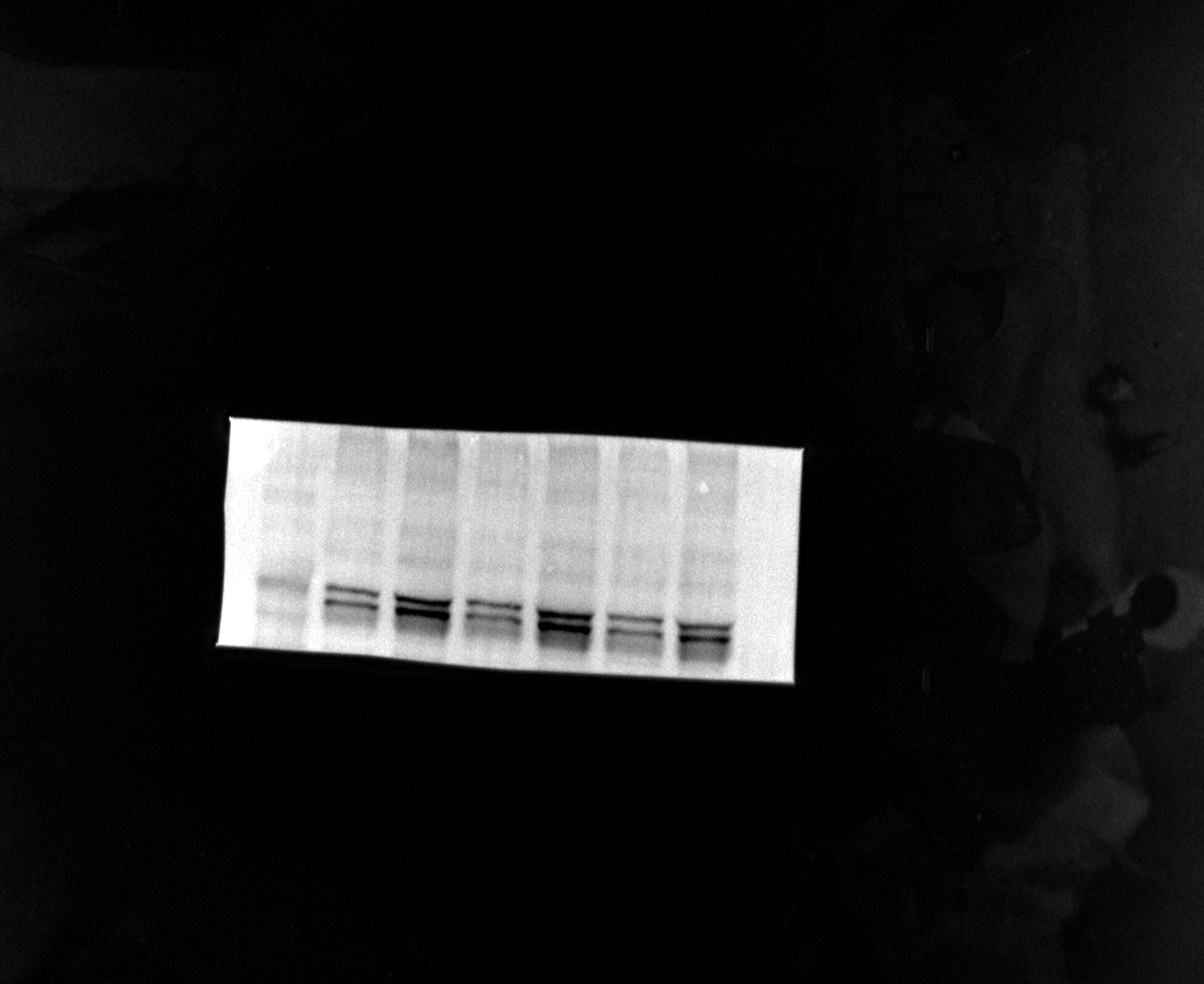

Supplement: Supplementary file 4 — Supplementary Information 4. [file 41598_2023_48990_MOESM4_ESM.tif]

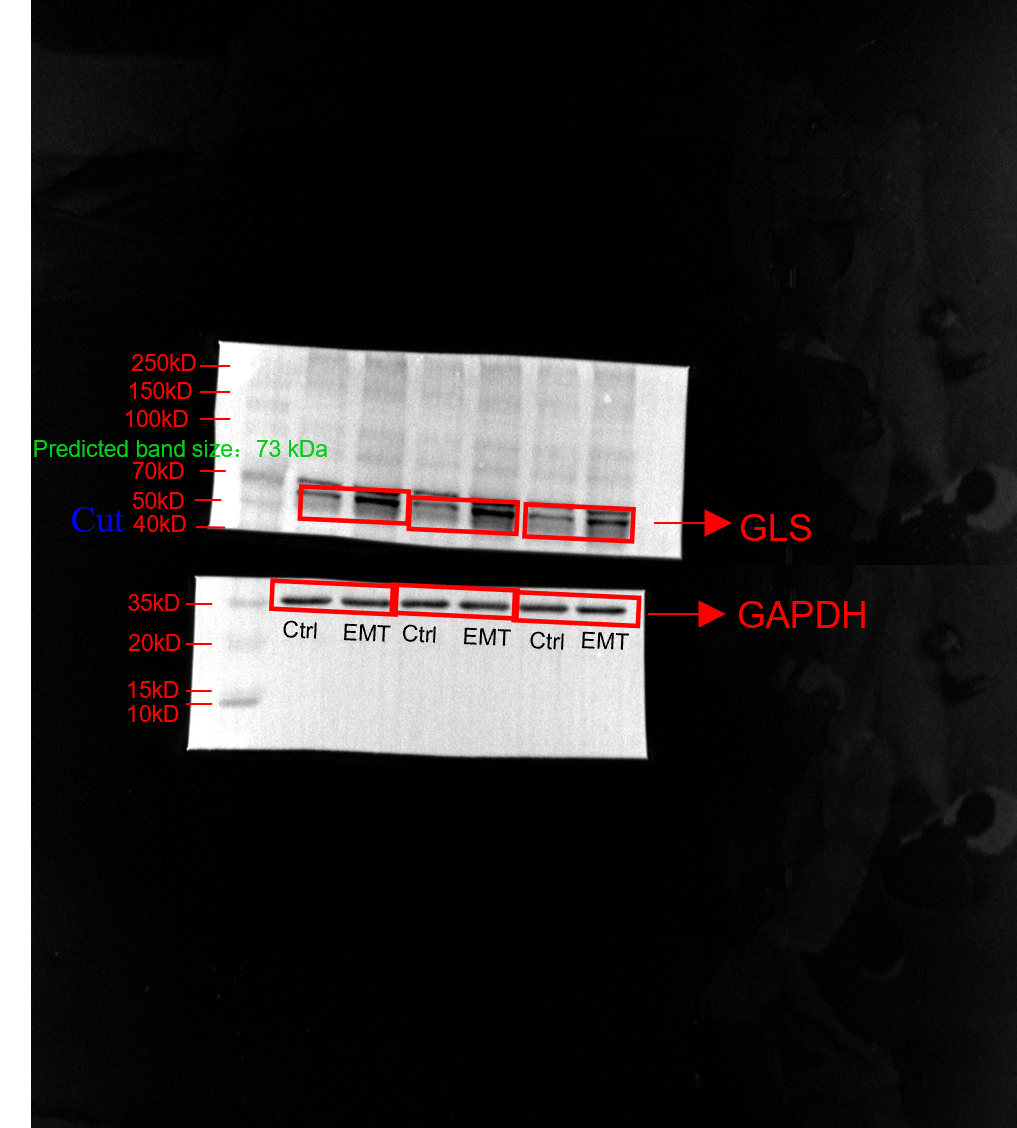

Supplement: Supplementary file 5 — Supplementary Information 5. [file 41598_2023_48990_MOESM5_ESM.png]

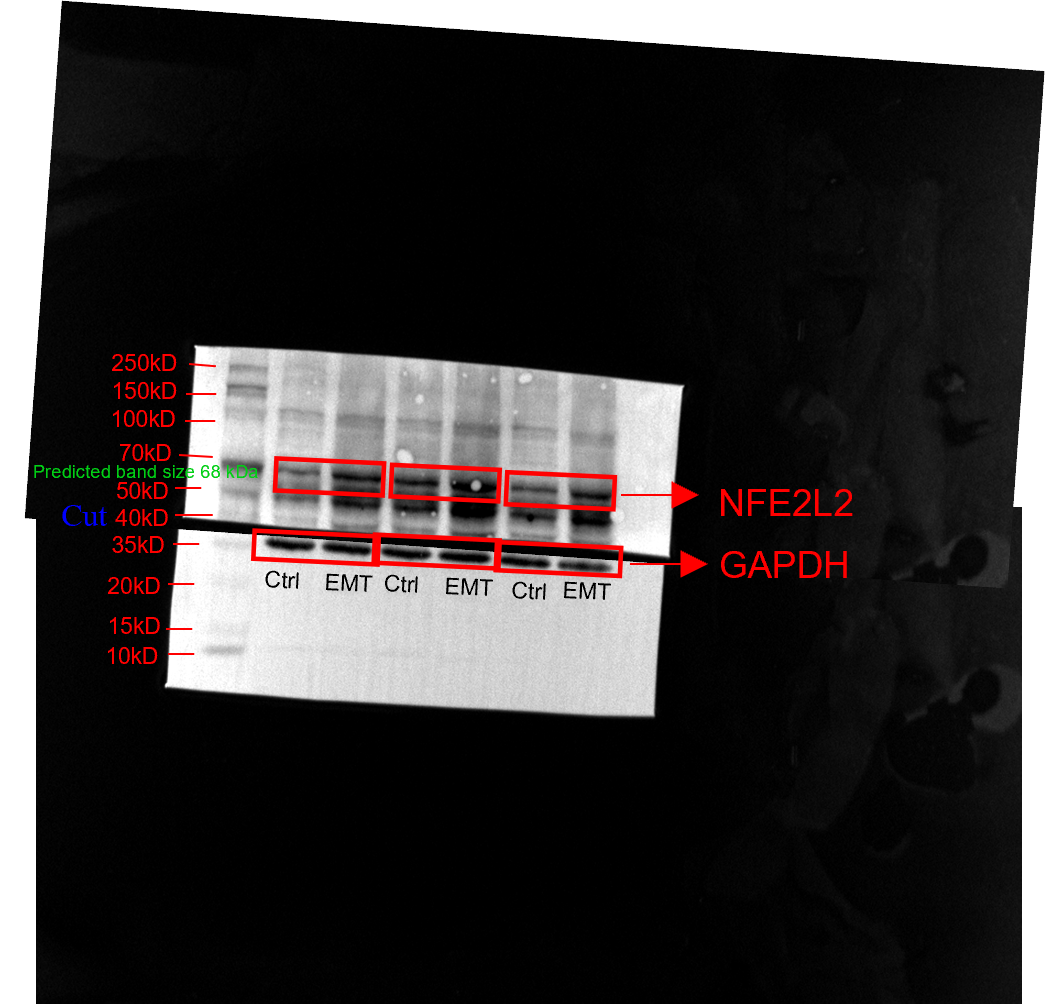

Supplement: Supplementary file 6 — Supplementary Information 6. [file 41598_2023_48990_MOESM6_ESM.png]

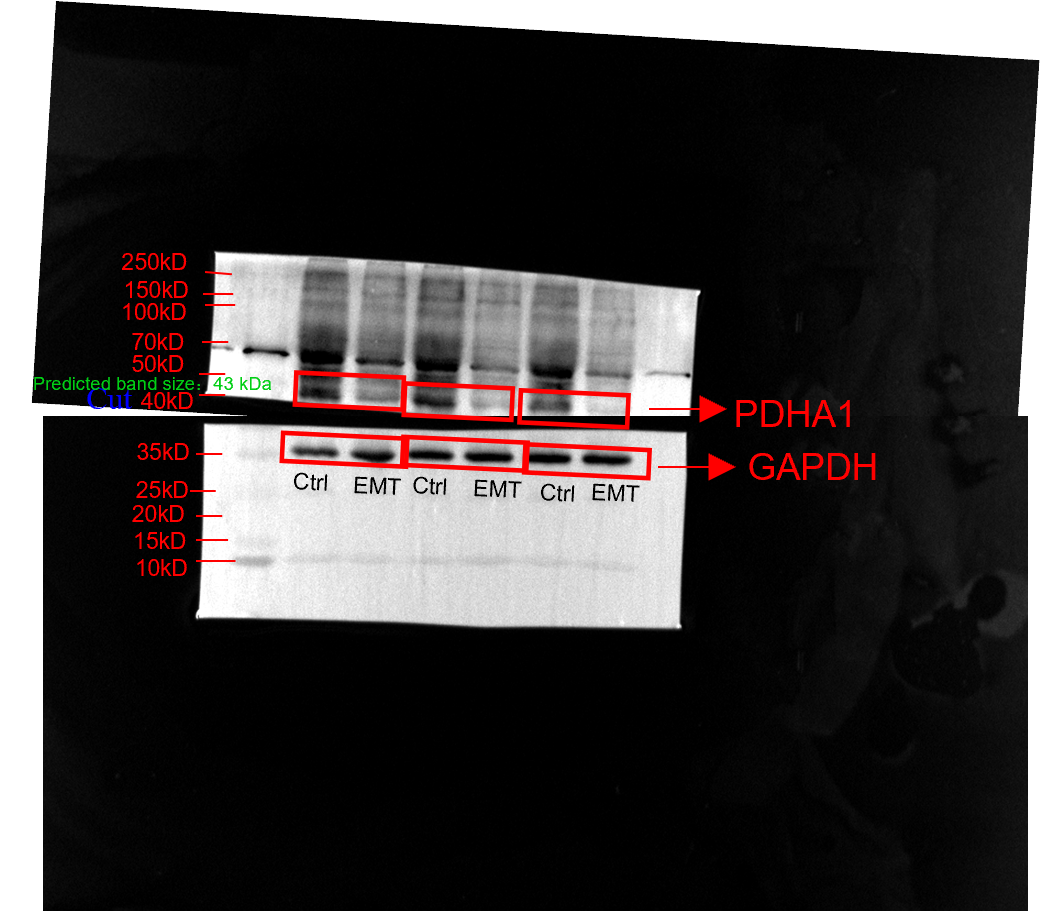

Supplement: Supplementary file 7 — Supplementary Information 7. [file 41598_2023_48990_MOESM7_ESM.png]

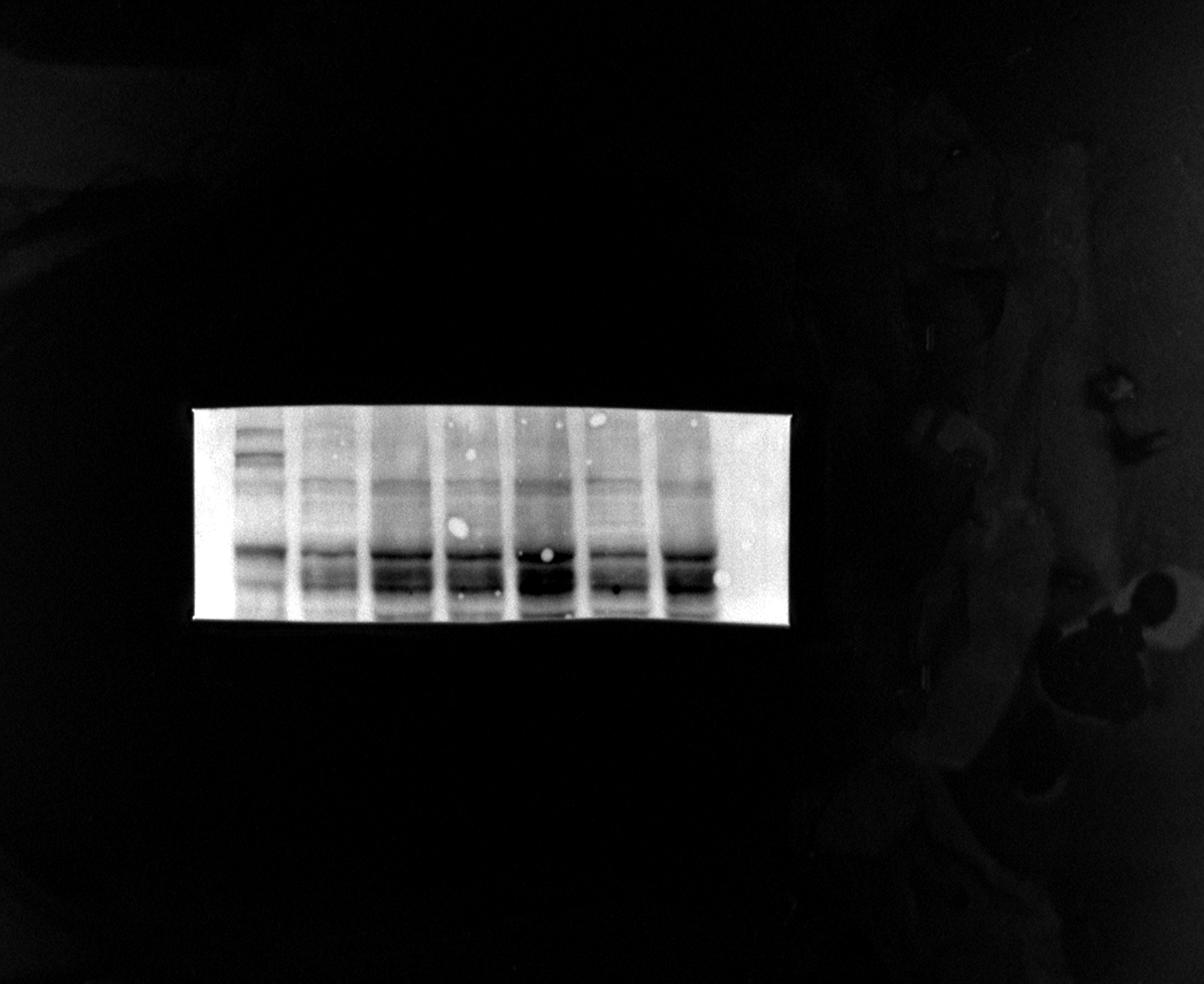

Supplement: Supplementary file 8 — Supplementary Information 8. [file 41598_2023_48990_MOESM8_ESM.tif]

A

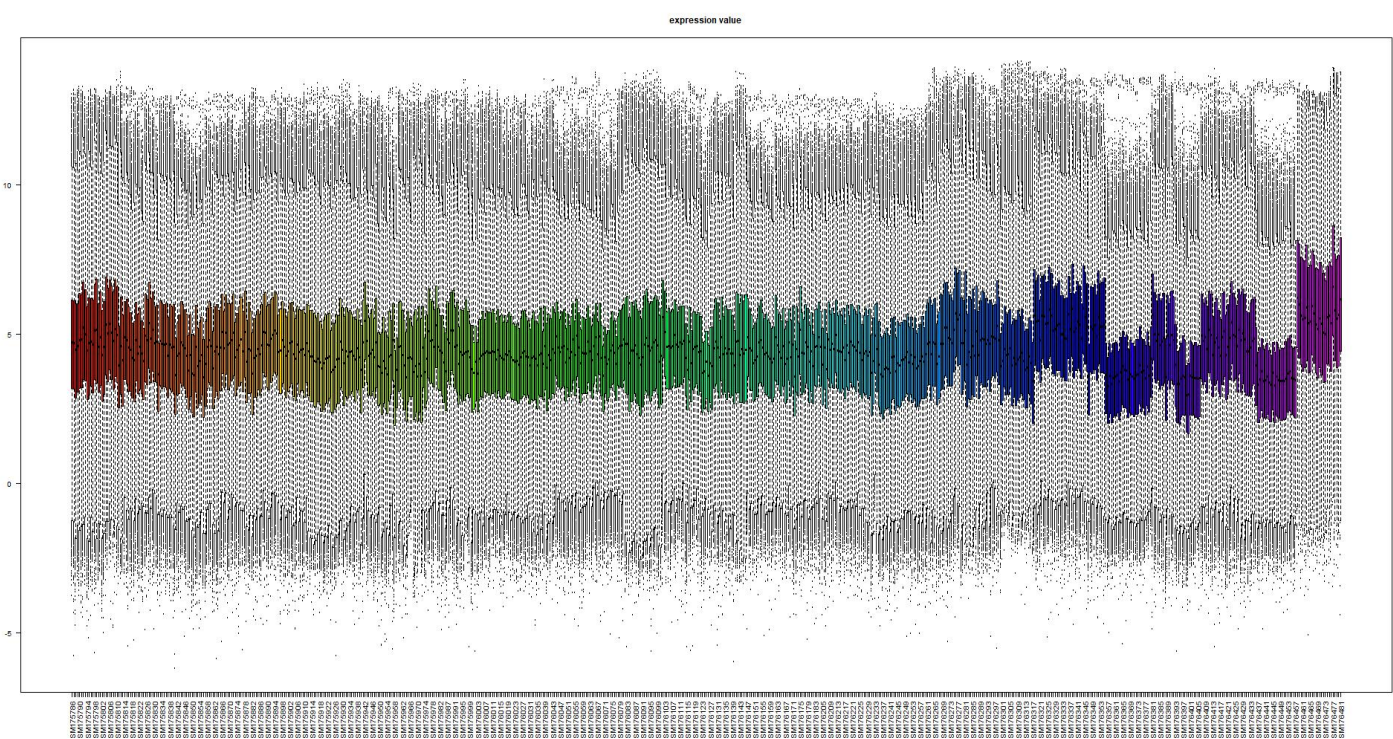

Before Normalization

B

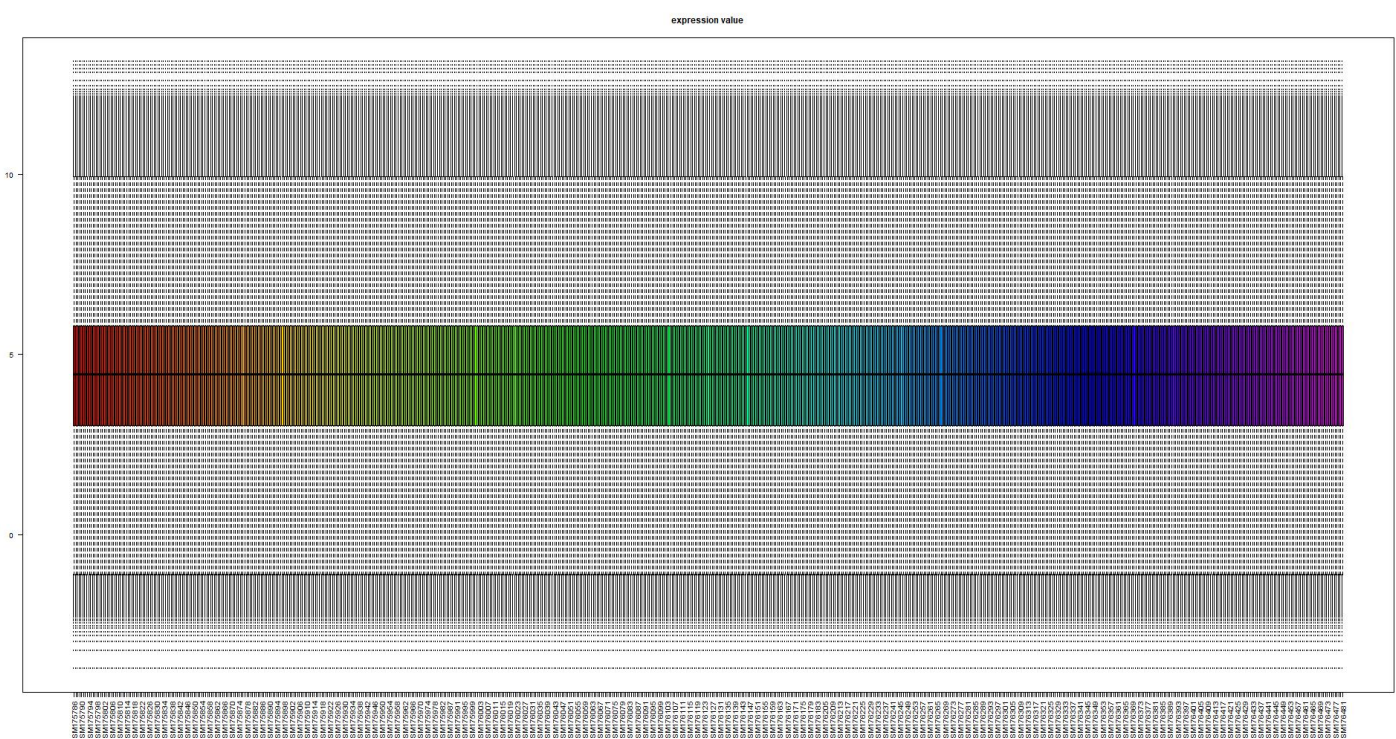

Normalization

C

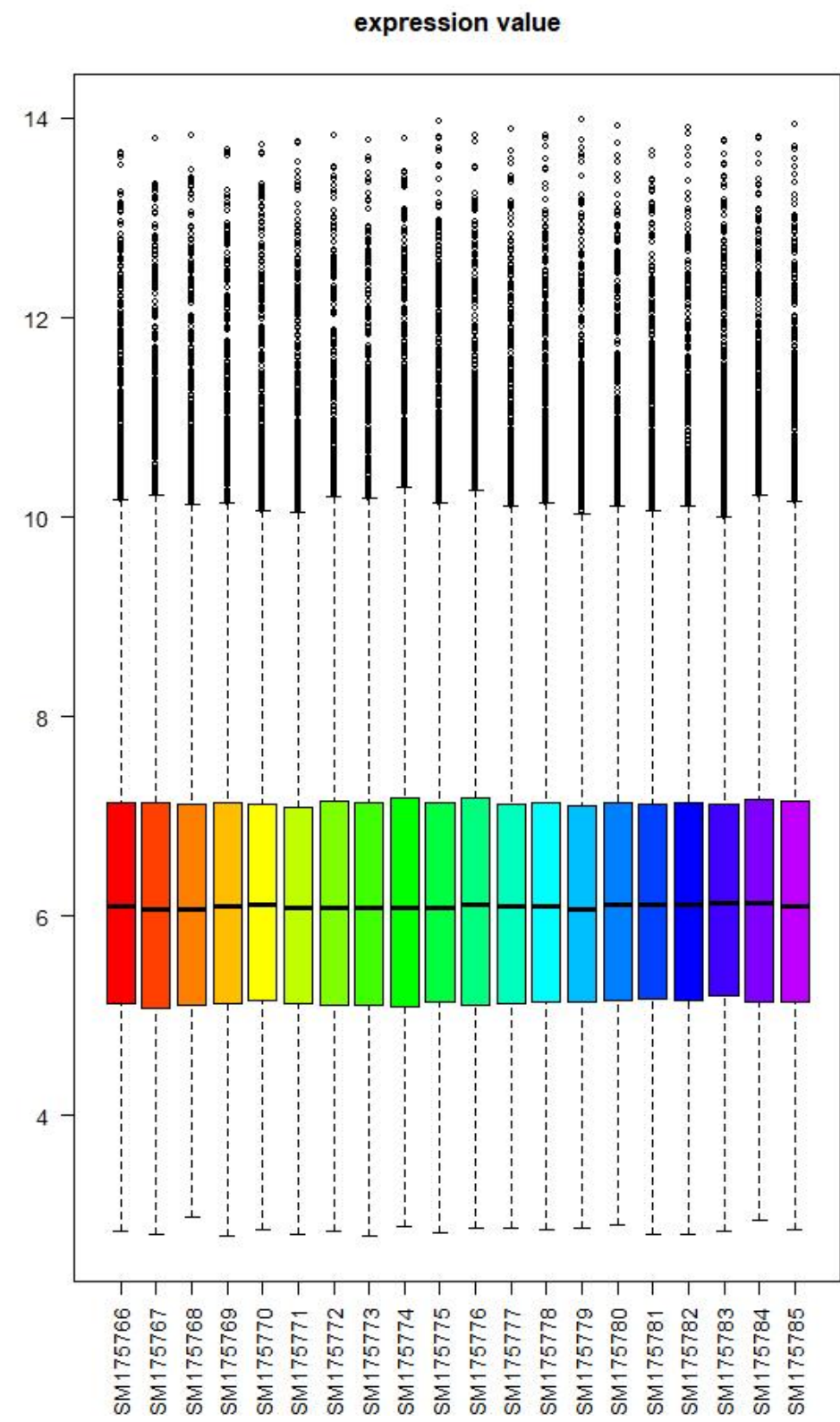

Before Normalization

D

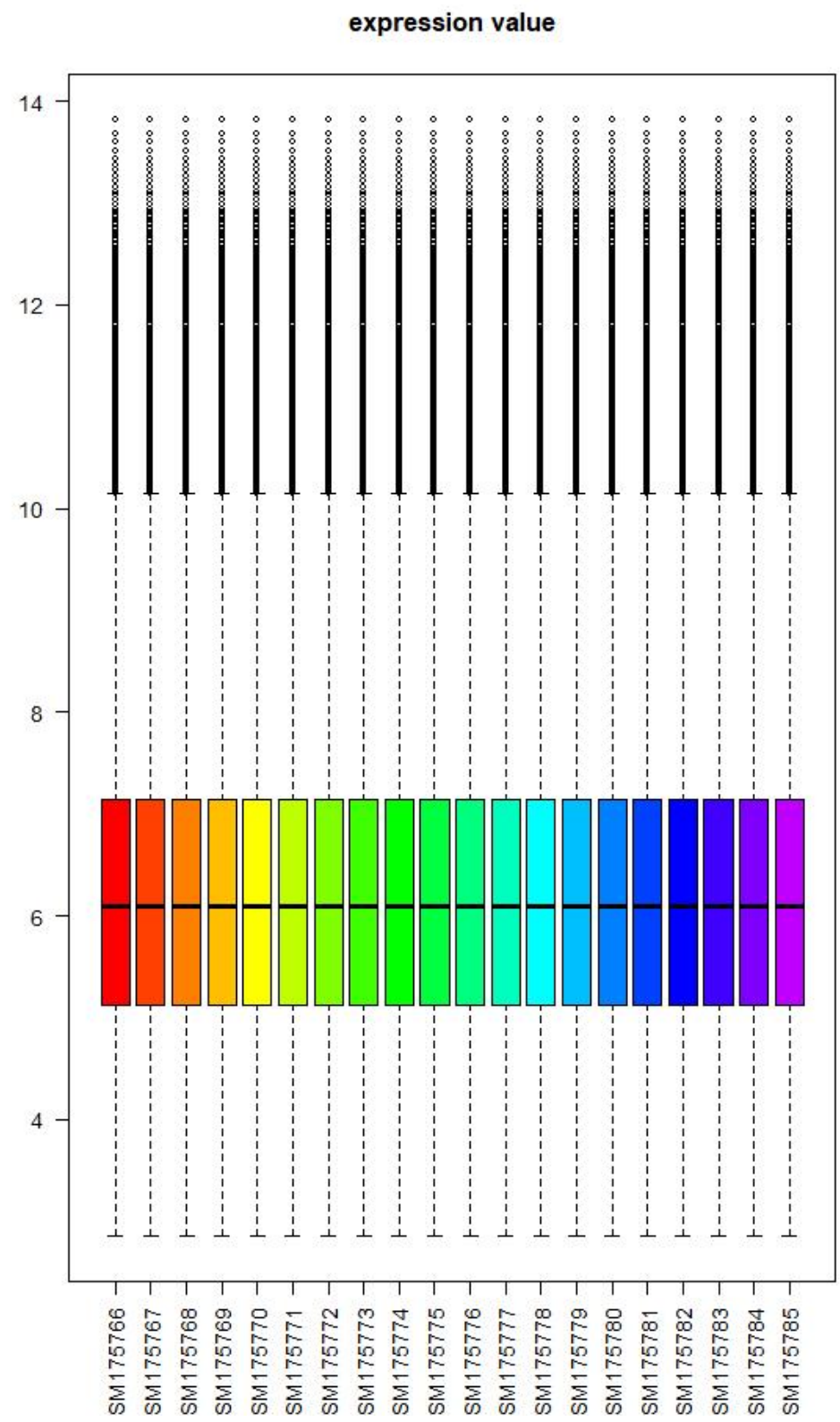

Normalization

Supplement: Supplementary file 9 — Supplementary Information 9. [file 41598_2023_48990_MOESM9_ESM.pdf]

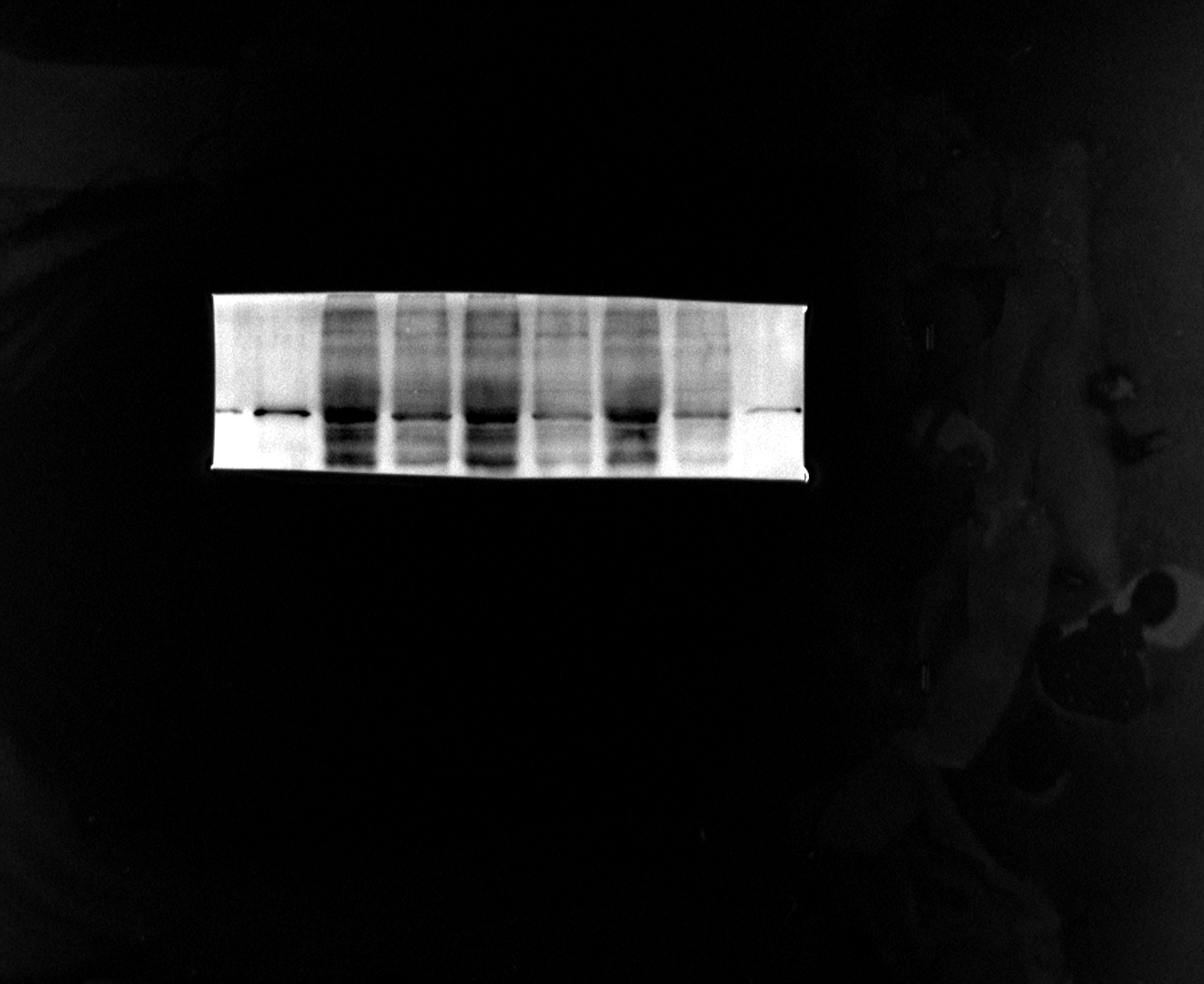

Supplement: Supplementary file 11 — Supplementary Information 11. [file 41598_2023_48990_MOESM11_ESM.tif]
