# Supplementary material for: Machine learning algorithms for a novel cuproptosis-related gene signature of diagnostic and immune infiltration in endometriosis
Source: Sci Rep. 2023 Dec 7;13:21603. doi: 10.1038/s41598-023-48990-w (PMC10703883; doi:10.1038/s41598-023-48990-w)
Supplement: Supplementary file 12 — Supplementary Information 12. [file 41598_2023_48990_MOESM12_ESM.docx]

| Gene | Sequences (5’ -3’ ) | Sequences (3’ -5’ ) |
| --- | --- | --- |
| GLS | CAGAAGGCACAGACATGGTTGG | GGCAGAAACCACCATTAGCCAG |
| NFE2L2 | CACATCCAGTCAGAAACCAGTGG | GGAATGTCTGCGCCAAAAGCTG |
| PDHA1 | GGATGGTGAACAGCAATCTTGCC | TCGCTGGAGTAGATGTGGTAGC |
| GAPDH | GAAAGCCTGCCGGTGACTAA | AGGAAAAGCATCACCCGGAG |

**Supplementary Table 1** Primers for Real-time PCR
